# Ferroptosis in retinal neurodegeneration: mechanistic vulnerability, therapeutic targeting, and translational barriers

**DOI:** 10.3389/fmed.2026.1890483

**Published:** 2026-09-16

**Authors:** Guangguang Wu, Suyu Wang, Ziyi Chen, Jiajun Li, Keran Li

**Affiliations:** Department of Ophthalmology, The Affiliated Eye Hospital, Nanjing Medical University, Nanjing, Jiangsu, China

**Keywords:** ferroptosis, lipid peroxidation, retinal neurodegeneration, targeted cross-barrier delivery, translational medicine

## Abstract

Progressive retinal neurodegeneration remains a major therapeutic challenge in ophthalmology because structural and functional loss may continue despite control of the primary insult, including intraocular pressure reduction in glaucoma, metabolic management in diabetic retinopathy, or suppression of acute inflammatory activity. Ferroptosis, an iron-dependent form of regulated cell death driven by phospholipid peroxidation and insufficient antioxidant buffering, has emerged as a potential mechanism linking metabolic stress, ischemic injury, excitotoxicity, and neuroinflammatory signaling to sustained retinal neurovascular unit damage. In this review, we synthesize current evidence supporting ferroptosis-related vulnerability in retinal neurodegenerative diseases. Particular emphasis is placed on expansion of the labile iron pool, PUFA-phospholipid remodeling, dysfunction of the System Xc⁻/glutathione (GSH)/glutathione peroxidase 4 (GPX4) axis, and multicellular amplification within the neuro-glial-microvascular unit. We further compare disease-specific evidence in glaucoma, diabetic retinopathy, and selected optic nerve injury contexts, emphasizing that causal validation remains uneven across models, cell types, and disease stages. From a translational perspective, we discuss why anti-ferroptotic strategies face distinct ophthalmic barriers, including limited retinal access, insufficient local exposure, short intraocular residence, and safety constraints associated with chronic modulation of iron and redox metabolism. Finally, we evaluate emerging enabling strategies, including nanocarrier-based delivery, long-acting gene modulation, and biomarker frameworks integrating ocular fluids, imaging, multi-omics, and artificial intelligence. Ferroptosis-targeted neuroprotection is therefore best viewed as a disease- and stage-dependent translational framework rather than a universal therapeutic solution.

## 1 Introduction

Retinal neurodegenerative diseases and optic neuropathies are major causes of irreversible visual impairment. Although these conditions possess distinct etiologies, their common outcome manifests as the progressive loss of retinal ganglion cells (RGCs) and their axons. Current therapies primarily target the primary pathological processes, such as lowering intraocular pressure (IOP) in glaucoma, metabolic management and anti-VEGF therapy in diabetic retinopathy (DR), and immunosuppression in optic neuritis (ON). However, even when these primary stressors are adequately controlled, secondary neurodegeneration often continues to progress (1). This phenomenon suggests the presence of underlying metabolic imbalances and the reprogramming of cell death pathways within the local microenvironment.

Histologically, the inner retina inherently exhibits a high susceptibility to ferroptosis. Retinal neurons operate under continuous high oxygen consumption and substantial mitochondrial loads within a microenvironment rich in polyunsaturated fatty acids (PUFAs) (2–4). Consequently, a delicate balance must be maintained among iron homeostasis, membrane lipid stability, and antioxidant defense systems. Under conditions of ischemia, excitotoxicity, chronic hyperglycemia, or inflammatory injury, this balance is readily disrupted, culminating in the expansion of the labile iron pool (LIP), the accumulation of membrane lipid peroxidation, and the failure of defense networks such as glutathione peroxidase 4 (GPX4) (3–7). Concurrently, microglial activation, Müller glia dysfunction, oligodendrocyte and myelin damage, and microvascular barrier abnormalities can further amplify this process (8–10). Therefore, within these representative pathological contexts, ferroptosis should be conceptualized as a critical downstream interface linking metabolic vulnerability and multicellular microenvironmental crosstalk to secondary neurodegeneration, rather than as a universal mechanism uniformly applicable across all optic neuropathies (11–13).

Building upon this mechanistic rationale, targeting ferroptosis has emerged as a compelling direction for ophthalmic neuroprotection research. In this review, we select glaucoma, DR, and inflammatory/demyelinating optic neuropathies as representative disease models to discuss how ferroptosis integrates into distinct upstream pathological drivers and to delineate its pathophysiological significance. Furthermore, we outline the potential translational value of cross-barrier delivery systems, long-term gene reprogramming, and artificial intelligence-assisted multi-omics biomarker frameworks. Ultimately, this review aims to critically evaluate the neuroprotective potential of ferroptosis-targeted interventions in retinal and optic nerve neurodegeneration from both mechanistic and translational perspectives.

## 2 Core mechanistic basis of ferroptosis in the retinal neurovascular unit and optic nerve axis

### 2.1 Metabolic vulnerability of the retina and dysfunction of the blood-retinal barrier

As direct extensions of the central nervous system, RGCs exhibit substantial metabolic demands. Their unmyelinated intraocular axons must continuously maintain ion gradients, axoplasmic transport, and action potential propagation, thereby relying heavily on mitochondrial oxidative phosphorylation (OXPHOS). Previous research indicates that the balance between energy supply and demand in RGCs presents an inherent metabolic vulnerability; under conditions of energy deprivation or oxidative stress, these cells are more prone to axonal dysfunction and cellular injury (3, 14, 15).

Beyond high energy requirements, retinal and optic nerve tissues possess a biochemical foundation that renders them susceptible to lipid peroxidation. Neural tissues are rich in PUFAs, and the bis-allylic structure of PUFAs makes them particularly vulnerable to free radical attack, serving as critical substrates for lipid peroxidation reactions (14, 16, 17). It is essential to emphasize that PUFA enrichment does not equate to the onset of ferroptosis itself; more accurately, this lipid composition heightens cellular susceptibility to ferroptosis when iron homeostasis is disrupted and antioxidant defenses are compromised (15, 18). The retina's profound reliance on lipid homeostasis has been corroboratively demonstrated in studies of retinal lipid metabolism.

Iron homeostasis is equally indispensable for normal RGC physiology. Iron participates in heme and iron-sulfur cluster synthesis and supports the function of proteins associated with the mitochondrial electron transport chain; consequently, neurons must maintain tightly regulated processes for iron uptake, utilization, and storage (14, 19, 20). However, under pathological conditions, dysregulation of iron metabolism can lead to the abnormal expansion of the LIP, which subsequently promotes Fe^2^⁺ participation in Fenton reactions, amplifying lipid peroxidation damage (19, 21, 22). Under stress conditions such as elevated IOP, ischemia, or excitotoxicity, disrupted axoplasmic transport and mitochondrial dysfunction in RGCs can further increase reactive oxygen species (ROS) production and exacerbate iron dyshomeostasis, thereby providing the necessary conditions for the initiation of ferroptosis (23–25).

It is important to note that the retina's susceptibility to ferroptosis is not solely determined by the intrinsic characteristics of neurons. Under physiological conditions, the blood-retinal barrier (BRB) coordinates with endothelial cells, pericytes, and the glial support network to maintain the local homeostasis of ions, metabolites, and inflammatory signals, while restricting the direct impact of peripheral noxious factors on neural tissue (26). The BRB also functions as a critical tissue barrier for retinal iron homeostasis. The presence of various iron-regulatory proteins in the retina suggests that local iron metabolism is not a passive process but rather is actively regulated. In addition to classical transferrin-associated iron transport, iron-import-related proteins such as Zip8 and Zip14 are expressed in the retina, indicating multi-layered regulation of local iron homeostasis; however, the actual contributions of these pathways in different pathological states and diverse cell types require further elucidation (27).

Under pathological conditions, compromised BRB function yields dual consequences. First, a decline in barrier integrity allows inflammatory mediators, oxidative stress, and iron metabolism abnormalities to more readily exert synergistic effects on RGCs, thereby lowering their tolerance threshold to ferroptotic stress (26). Second, damage to the BRB and associated microvascular structures signifies that the homeostasis of the retinal microenvironment itself is shifting—a process not restricted to neurons alone. Recent studies suggest that in models of DR, retinal microvascular cells may exhibit ferroptosis-related alterations, which are associated with early microvascular dysfunction; nevertheless, the cell-type specificity and causal role of pericyte ferroptosis across different retinal diseases necessitate further direct evidentiary support (28, 29).

Therefore, the susceptibility of the retina to ferroptosis reflects a layered vulnerability of the retinal neurovascular unit. The high energy demand, PUFA-rich membrane composition, and iron-dependent metabolism of RGCs provide the intracellular basis for ferroptosis-prone injury, whereas BRB integrity and microvascular-glial support determine whether this vulnerability is amplified under disease conditions. This interpretation is important because it shifts the focus from neuron-intrinsic biochemical stress to a compartment-specific injury environment. It also explains why effective ferroptosis-targeted neuroprotection may require not only molecular inhibition of lipid peroxidation, but also strategies that preserve barrier function, improve local delivery, and stabilize the surrounding glial-microvascular niche.

### 2.2 Retinal biochemical thresholds for ferroptosis susceptibility

Ferroptosis-related biochemical pathways are most relevant to retinal neurodegeneration when they explain how retinal cells cross the threshold from reversible oxidative stress to membrane-destructive injury. In this context, the purpose of this section is not to provide a comprehensive account of ferroptosis biology, but to define the major cell-intrinsic conditions that make RGCs, glial cells, and microvascular components more susceptible to iron-dependent lipid peroxidation. These conditions mainly include redox-active iron availability, peroxidizable membrane lipid substrates, and insufficient lipid peroxide detoxification. Together, they provide a compact biochemical framework linking retinal metabolic vulnerability to ferroptosis-prone injury.

Iron dyshomeostasis is a critical determinant of this threshold. RGCs rely heavily on mitochondrial oxidative metabolism, axonal maintenance, heme synthesis, and iron-sulfur cluster-dependent enzymatic activity; therefore, retinal neurons require tightly regulated iron uptake, utilization, storage, and mobilization (14, 19, 20, 22). Under pathological conditions, the relevant event is not simply an increase in total iron, but the expansion of the labile iron pool (LIP), which increases the availability of redox-active iron and facilitates lipid peroxidation (19, 21, 22). This distinction is important for retinal disease because RGCs already operate under high metabolic demand and continuous oxidative pressure. Once local iron handling is disturbed, their tolerance to additional ischemic, excitotoxic, pressure-related, or metabolic stress may decline rapidly.

Ferritinophagy provides one plausible route through which stored iron can be mobilized into a reactive pool. Foundational studies identifying nuclear receptor coactivator 4 (NCOA4) as a cargo receptor for ferritinophagy support this mechanism at the general cell-biological level (30, 31). In ophthalmic research, elevated intraocular pressure has been shown to induce retinal Fe^2^⁺ accumulation, ferritin degradation, and NCOA4-associated ferritinophagy, whereas inhibition of this process can attenuate RGC ferroptosis-related injury (32). Therefore, in this section, NCOA4-mediated ferritinophagy should not be presented as an independent mechanistic branch in detail, but as a retinally relevant mechanism that may explain how pressure-related or ischemic stress expands redox-active iron availability in vulnerable RGCs.

The second determinant is whether retinal cells contain sufficient peroxidizable membrane substrates. Neural and glial membranes in the retina and optic nerve are enriched in polyunsaturated fatty acids (PUFAs), which support membrane fluidity, receptor function, and neural signal transduction, but also increase susceptibility to phospholipid peroxidation under oxidative stress (2, 16, 17, 33). PUFA enrichment alone does not initiate ferroptosis; rather, it increases vulnerability when iron dyshomeostasis and insufficient antioxidant defense coexist (15, 18). Enzymes such as ACSL4 and LPCAT3 further shape ferroptosis sensitivity by promoting the incorporation of PUFAs into membrane phospholipids (16, 18, 34). For retinal neurodegeneration, the key point is not to fully expand the enzymatic lipid-remodeling pathway, but to emphasize that this substrate-forming process may make RGCs and associated retinal cells more permissive to iron-dependent lipid peroxidation.

This lipid substrate framework helps connect general ferroptosis biology to retinal pathology. The LIP expansion discussed above provides catalytic conditions for iron-dependent oxidation, whereas ACSL4/LPCAT3-associated lipid remodeling determines whether oxidative stress can continuously act on susceptible PUFA-containing membranes (35). In high intraocular pressure-related injury, increased retinal ACSL4 expression has been associated with iron dyshomeostasis and RGC damage, suggesting that lipid remodeling may cooperate with iron stress to promote ferroptosis-related injury. However, the causal contribution of this pathway across retinal cell types and disease stages remains incompletely defined. Therefore, lipid metabolism remodeling should be framed as a susceptibility-modifying process, rather than as a fully established retinal disease driver.

The third determinant is the capacity of retinal cells to detoxify lipid peroxides once they are formed. The System Xc⁻/glutathione (GSH)/glutathione peroxidase 4 (GPX4) axis represents the canonical defense pathway against phospholipid hydroperoxide accumulation (4, 5). In the retina, this defense system is closely linked to neuron-glia metabolic coupling and glutamate homeostasis. Under pathological conditions such as glaucoma, ischemia-reperfusion injury, or glial dysfunction, extracellular glutamate accumulation may restrict System Xc⁻-dependent cystine uptake, reduce GSH synthesis, and weaken GPX4-mediated lipid peroxide clearance (13, 36–39). Evidence from GLAST-deficient models further supports the connection between impaired glutamate handling, RGC loss, and ferroptosis-related molecular alterations (36, 39). Thus, antioxidant defense failure in retinal neurodegeneration should not be interpreted only as a generic decline in redox capacity, but as a consequence of disrupted metabolic support within the retinal microenvironment.

Parallel defense systems may provide additional buffering, but they should remain secondary in this section. FSP1-CoQ10, mitochondrial DHODH, and NRF2-regulated antioxidant programs can counteract lipid peroxidation through mechanisms complementary to the GPX4-GSH axis (7, 40, 41). However, their retinal cell-type specificity and disease-stage relevance remain less clearly defined than the central GPX4-associated defense framework. For this reason, these pathways are best positioned as auxiliary protective systems that may modify ferroptosis susceptibility when GPX4-dependent protection becomes insufficient, rather than as separate mechanisms requiring extended discussion in this chapter.

Importantly, the molecular determinants of ferroptosis susceptibility also perform essential physiological functions in the normal retina. Iron is required for heme and iron–sulfur cluster synthesis, mitochondrial respiration, and other iron-dependent enzymatic processes, whereas PUFA-containing phospholipids contribute to membrane organization, fluidity, and neural signaling. Similarly, low levels of reactive oxygen species participate in physiological redox signaling, while the GSH/GPX4 and complementary antioxidant systems maintain lipid peroxide concentrations within a tolerable range rather than eliminating oxidative signaling altogether. Ferroptosis therefore emerges when these normally homeostatic processes become quantitatively or spatially dysregulated, rather than from the mere presence of iron, PUFAs, or ROS. This distinction is important therapeutically because excessive suppression of these pathways may itself disturb retinal metabolic and membrane homeostasis.

Taken together, redox-active iron availability, peroxidizable membrane lipid substrates, and lipid peroxide detoxification capacity define the biochemical threshold for ferroptosis susceptibility in retinal cells. These processes should not be treated as isolated molecular pathways. Instead, they explain how retinal metabolic stress, pressure-related injury, diabetic redox imbalance, ischemia, or inflammatory damage may converge on iron-dependent lipid peroxidation. This cell-intrinsic biochemical threshold provides the link between the tissue-level vulnerability described above and the neuro-glial-microvascular amplification discussed in the following section. These interconnected biochemical processes are summarized in Figure 1.

**Figure 1:**
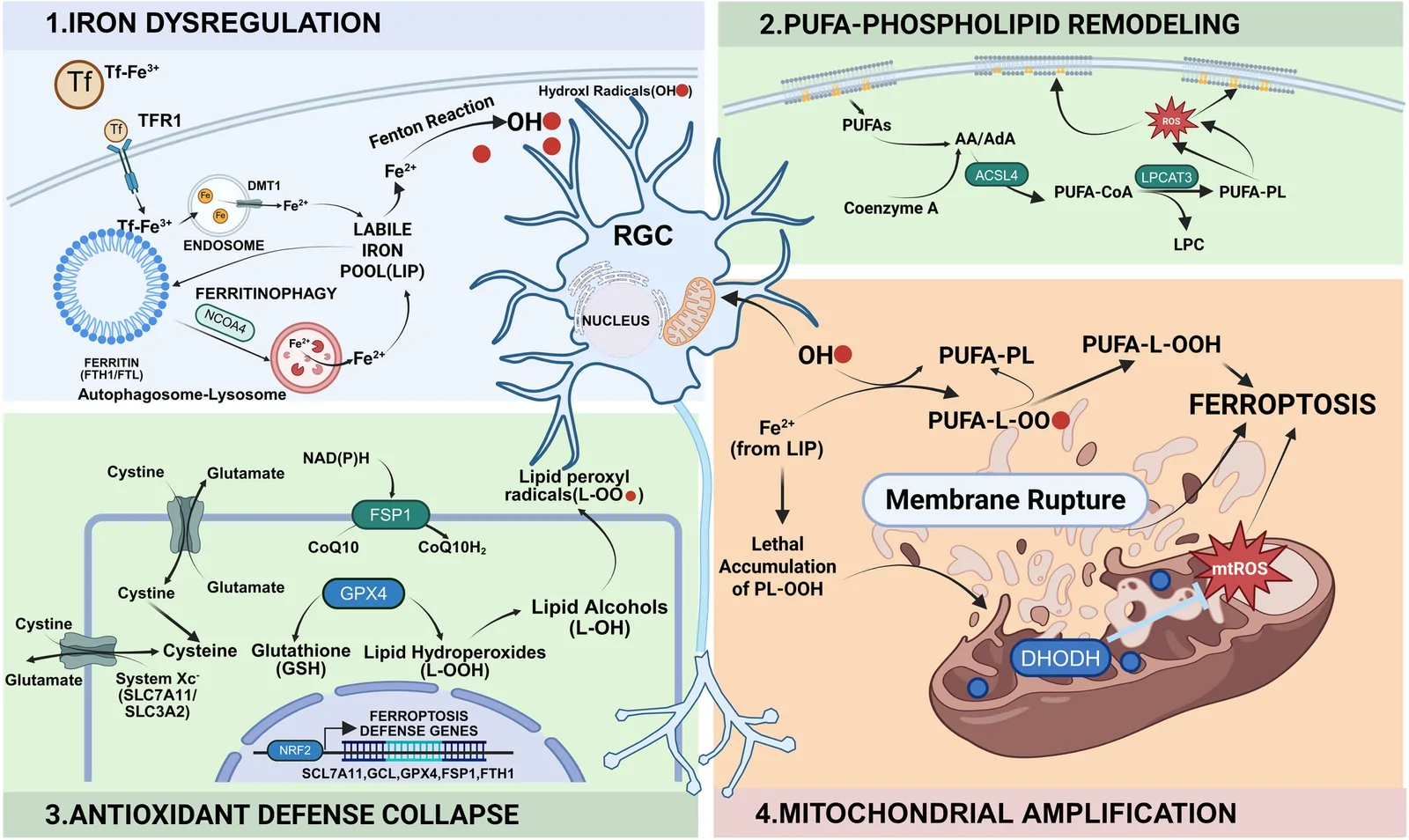
Core biochemical network and intrinsic metabolic susceptibility of ferroptosis in RGCs. RGCs are susceptible to ferroptosis because of high energy demand, mitochondrial dependence, PUFA-rich membranes, and iron-dependent metabolism. Iron dyshomeostasis expands the labile iron pool through altered iron uptake and NCOA4-mediated ferritinophagy, while ACSL4/LPCAT3-mediated lipid remodeling increases the pool of peroxidizable PUFA-phospholipids. The System Xc⁻/GSH/GPX4 axis provides the canonical defense against phospholipid hydroperoxides, with FSP1-CoQ10, mitochondrial DHODH, and NRF2-regulated programs serving as complementary protective systems. Failure of these defenses under sustained stress promotes irreversible ferroptotic injury. Created with http://biorender.com

### 2.3 Neuro-glial network communication and the dysregulation of ferroptotic home-ostasis in the retinal microenvironment

Ferroptosis of RGCs is strongly influenced by the surrounding neuro-glial-microvascular network. Pathological stressors first disturb the local retinal microenvironment, and this disturbance can lower the ferroptosis threshold of RGCs through inflammatory signaling, glutamate dysmetabolism, iron redistribution, oxidative stress, and barrier dysfunction (8–10, 42). In this setting, ferroptosis is not only a terminal mode of cell death, but also a useful framework for understanding how multicellular crosstalk converts heterogeneous upstream insults into sustained neurodegenerative injury.

In this process, microglia are typically among the first components to become activated. Under physiological conditions, microglia are responsible for local surveillance and homeostasis maintenance. However, under conditions of elevated IOP, ischemia, or inflammatory stimuli, their phenotype shifts toward a pro-inflammatory state, releasing cytokines such as tumor necrosis factor alpha (TNF-α), interleukin-1 beta (IL-1β), and interleukin-6 (IL-6), while simultaneously increasing local ROS through NADPH oxidase 2 (NOX2) mediation. These alterations directly augment the oxidative burden surrounding neurons and concomitantly disrupt iron transport equilibrium, rendering RGCs more susceptible to intracellular reactive iron accumulation (12, 43–46). Notably, microglia themselves exhibit a relatively high susceptibility to ferroptosis; should they undergo ferroptotic injury, the subsequent release of intracellular iron and damage-associated molecular patterns (DAMPs) further amplifies local inflammation and oxidative stress (9, 47, 48). This does not constitute a single insult, but rather establishes a pathological amplification loop capable of continuously driving the expansion of injury. This amplification loop suggests that microglia-related ferroptosis is relevant not only as a source of inflammatory injury, but also as a potential target for interrupting the spread of iron-dependent oxidative stress within the retinal microenvironment.

Accompanying this is the functional failure of the glial support system. Astrocytes and Müller glia within the retina collectively participate in maintaining glutamate homeostasis, a function that is often compromised early in chronic pathological states. Following a decline in the expression of glutamate transporters such as glutamate/aspartate transporter (GLAST) and glutamate transporter-1 (GLT-1), extracellular glutamate gradually accumulates. This not only exacerbates excitotoxicity but also inhibits System Xc⁻-mediated cystine uptake, leading to deficient GSH synthesis and an impaired GPX4 defense network (13, 36–38). In this regard, glial dysfunction is a necessary component in the formation of RGC ferroptosis. Previous studies using GLAST-deficient models have indicated that impaired glutamate clearance alone can induce continuous RGC loss accompanied by ferroptosis-related molecular alterations, a process that can be partially mitigated by ferroptosis inhibitors (36, 39). Thus, glial glutamate-handling failure provides a mechanistic bridge between excitotoxicity and ferroptosis, and may help identify disease contexts in which reinforcement of antioxidant buffering alone is insufficient without restoration of glial metabolic support.

Extending the focus from the inner retina to the myelinated regions of the optic nerve, the importance of oligodendrocytes and myelin structures becomes evident. Oligodendrocytes themselves constitute one of the cell populations with the highest iron content in the central nervous system, and their metabolic state is directly related to myelin maintenance and axonal conduction. During demyelination or inflammatory injury, myelin degradation can lead to the concurrent release of stored iron and lipid-rich membrane components, thereby simultaneously providing the local environment with both the catalytic and substrate conditions requisite for ferroptosis (8, 11, 49–55). Consequently, in pathologies such as optic neuritis, oligodendrocyte damage is not merely a sequela of axonal degeneration; it may itself serve as a source promoting the subsequent development of ferroptosis. This layer of significance expands the scope of ferroptosis beyond the retinal neurons themselves, linking it to glial-axonal interactions along the entire length of the optic nerve.

Collectively, ferroptosis-related injury in retinal and optic nerve neurodegeneration emerges from network-level disequilibrium rather than from a neuron-autonomous process alone. Pro-inflammatory microglial activation, impaired Müller glia and astrocyte support, oligodendrocyte and myelin injury, and BRB/microvascular dysfunction converge to sustain iron accumulation, lipid peroxidation, and insufficient antioxidant defense. This multicellular framework is important for two reasons. First, it explains why ferroptosis may contribute differently across glaucoma, diabetic retinopathy, and inflammatory or demyelinating optic neuropathies. Second, it indicates that therapeutic translation should prioritize compartment-matched strategies capable of stabilizing the ferroptosis-prone microenvironment, rather than relying only on free small-molecule inhibitors. This feed-forward injury network is summarized in Figure 2, and the major cellular contributors are listed in Table 1.

**Figure 2:**
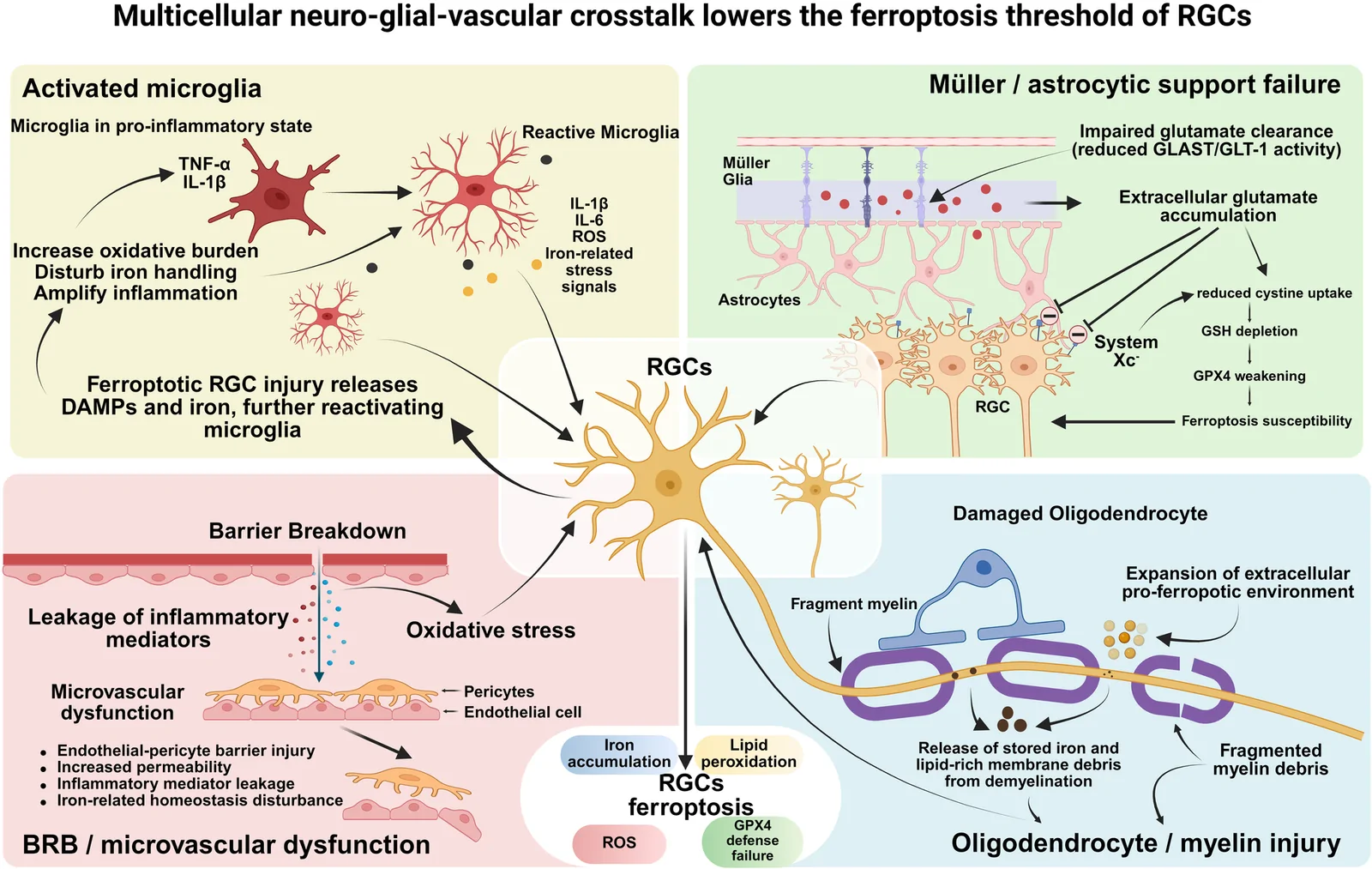
Neuro-glial-microvascular crosstalk shapes a ferroptosis-prone microenvironment for RGCs. In retinal and optic nerve neurodegeneration, activated micro-glia increase inflammatory and oxidative stress, Müller glia and astrocyte dysfunction impair glutamate clearance and antioxidant buffering, oligodendrocyte and myelin injury provide iron and lipid-rich stress signals, and BRB/microvascular disruption increases exposure to inflammatory and metabolic insults. Together, these processes create a ferroptosis-prone microenvironment in which RGC injury may further release DAMPs, iron, and lipid peroxidation products, sustaining a feed-forward injury loop. Created with http://biorender.com

**Table 1:** Major cellular contributors to a ferroptosis-prone microenvironment in retinal and optic nerve neurodegeneration.

Cell type	Major pathological change	Ferroptosis-relevant contribution	Main downstream consequence
RGCs	Axonal transport failure; mitochondrial dysfunction; redox imbalance	LIP expansion, lipid peroxidation, and weakened antioxidant defense	Soma and axonal degeneration, with release of DAMPs and lipid peroxidation byproducts
Microglia	Pro-inflammatory activation and oxidative stress	Release of inflammatory mediators and ROS; amplification of iron-related and inflammatory injury	Exacerbation of local oxidative stress and ferroptosis susceptibility
Müller glia and astrocytes	Impaired glutamate clearance; reduced metabolic and antioxidant support	Suppression of System Xc⁻-dependent defense and reduced GSH/GPX4 buffering	Increased excitotoxic and ferroptotic stress in adjacent RGCs
Oligodendrocytes	Demyelination and loss of axonal metabolic support	Release of stored iron and lipid-rich substrates that favor ferroptotic injury	Promotion of a ferroptosis-permissive microenvironment in the myelinated optic nerve
Microvascular cells	BRB dysfunction and microvascular instability	Amplification of inflammatory, oxidative, and iron-related disturbances	Indirect increase in RGC vulnerability through neurovascular unit disequilibrium

This table summarizes how distinct cellular components collectively shape a ferroptosis-prone microenvironment, rather than listing isolated cell death modes.

## 3 Disease-Specific drivers of ferroptosis in retinal neurodegeneration and optic neuropathies

Retinal neurodegenerative diseases and optic neuropathies exhibit substantial etiological heterogeneity. Glaucoma is primarily associated with intraocular pressure-related biomechanical stress, impaired axonal transport, and local ischemic dysregulation. Diabetic retinopathy (DR) develops in a chronic metabolic and microvascular environment shaped by hyperglycemia, oxidative stress, inflammation, and blood-retinal barrier (BRB) dysfunction. Optic neuritis (ON) and demyelinating optic neuropathies are driven mainly by immune-mediated inflammation, oligodendrocyte injury, demyelination, and subsequent axon-glia uncoupling. These differences should be preserved when evaluating ferroptosis-related mechanisms.

Across these disease contexts, ferroptosis may help explain how distinct primary insults are converted into sustained retinal ganglion cell (RGC) or axonal injury through iron dyshomeostasis, lipid peroxidation, and insufficient antioxidant defense. This convergence, however, does not imply that ferroptosis has the same weight or timing in each disease. In the following sections, glaucoma, DR, and ON-related demyelinating injury are discussed as representative settings in which ferroptosis-related mechanisms intersect with disease-specific pathology.

### 3.1 Glaucoma

The pathological hallmark of glaucoma is the progressive degeneration of RGCs and their axons; however, this process cannot be explained solely by mechanical injury secondary to elevated IOP. Although IOP lowering remains the cornerstone of current treatment, structural and functional deterioration may still progress in a subset of patients, including those with normal-tension glaucoma (NTG) (56–58). This clinical observation suggests that glaucomatous neurodegeneration is not determined exclusively by the primary mechanical insult, but also involves secondary injury processes within the local retinal and optic nerve microenvironment. Within this context, ferroptosis should be viewed as a plausible downstream contributor to sustained neuronal damage rather than as a fully established universal mechanism in glaucoma.

Major upstream stressors in glaucoma include chronic IOP-related biomechanical strain, impaired microcirculation at the lamina cribrosa, and secondary ischemic or reperfusion-associated injury. In pressure-related disease, the lamina cribrosa represents an early site of tissue vulnerability. Persistent IOP elevation can distort this structure, compromise local perfusion, and disrupt axoplasmic transport in RGC axons. These events are accompanied by mitochondrial dysfunction and increased production of mitochondrial ROS, while fluctuating perfusion may further amplify oxidative burden (59, 60). Accordingly, the primary insult in glaucoma is better understood as a composite stress state involving mechanical deformation, mitochondrial stress, and ischemic dysregulation rather than simple pressure-mediated compression alone.

Against this background, ferroptosis-relevant mechanisms may help explain how initial glaucomatous stress is translated into sustained neurodegeneration. Experimental studies suggest that pressure-related injury can be associated with iron dyshomeostasis, expansion of the LIP, lipid peroxidation, and weakening of antioxidant defense systems (32). In parallel, impairment of glial glutamate handling may further lower the ferroptosis threshold: extracellular glutamate accumulation can restrict System Xc⁻-dependent cystine uptake, limit glutathione synthesis, and weaken GPX4-associated protection, while excitotoxic signaling may exacerbate oxidative and iron-related imbalance (13, 36–38, 61). Taken together, these findings support the view that ferroptosis may function as a downstream interface through which mechanical stress, ischemia, and excitotoxicity converge within the glaucomatous microenvironment.

This process is unlikely to be restricted to RGCs alone. Müller glia exposed to oxidative stress may also develop ferroptosis-related alterations, and glial dysfunction may secondarily amplify neuronal vulnerability through the release of inflammatory mediators and lipid peroxidation byproducts such as 4-hydroxynonenal (4-HNE) (62–64). Thus, in glaucoma, ferroptosis is more appropriately framed as part of a multicellular injury network than as an isolated neuron-intrinsic event. This interpretation is consistent with the broader view that glaucomatous progression reflects persistent dysregulation of the neuro-glial microenvironment (65).

From a translational standpoint, experimental glaucoma and optic nerve injury currently provide some of the strongest preclinical support for ferroptosis-targeted neuroprotection. *In vivo* studies have linked elevated intraocular pressure to iron dyshomeostasis, NCOA4-associated ferritinophagy, lipid peroxidation, and RGC injury, while interference with ferroptosis-related iron mobilization attenuates neuronal damage. Pharmacological inhibition with ferroptosis-suppressing agents such as Ferrostatin-1 has further been associated with improved RGC survival in experimental optic neuropathy models. Importantly, genetic reinforcement of ferroptosis defense through RGC-directed AAV-mediated GPX4 overexpression has also produced neuroprotective and regeneration-related effects. The convergence of biochemical evidence, pharmacological rescue, and genetic modulation strengthens the causal argument for ferroptosis in these models. Nevertheless, pharmacological rescue alone cannot establish pathway specificity because antioxidant and iron-modulating agents may also influence ferroptosis-independent oxidative injury, and long-term validation in chronic glaucoma models remains limited (39, 66).

### 3.2 Diabetic retinopathy

DR has traditionally been regarded primarily as a microvascular disease, with clinical attention focused on vascular leakage, neovascularization, and macular edema. However, this framework is incomplete. Although anti-vascular endothelial growth factor (VEGF) therapy and systemic glycemic control can improve major vascular manifestations, they often fail to reverse neural and glial abnormalities that may already be present at earlier stages of disease. Increasing evidence suggests that functional impairment and structural damage within the inner retina can precede overt microvascular lesions, including early changes involving retinal neurons and Müller glia (67, 68). Recent evidence further suggests a bidirectional interaction between early retinal inflammation and neurodegeneration, potentially forming a pathological amplification loop before advanced vascular manifestations become evident (69). Accordingly, DR is better understood not simply as an isolated vascular disorder, but as a chronic neurovascular unit disease in which neuronal, glial, and microvascular dysfunction evolve in parallel.

The predominant upstream stressors in DR include chronic hyperglycemia, sustained metabolic overload, and persistent oxidative and inflammatory stress. Under high-glucose conditions, retinal cells are exposed to prolonged mitochondrial burden, which promotes electron leakage from the respiratory chain and excessive generation of mitochondrial ROS. In parallel, hyperglycemia can activate NOX-related oxidative pathways, amplify inflammatory signaling, and compromise BRB integrity (70–74). Thus, the primary pathological background in DR is more accurately defined as a chronic state of metabolic and redox disequilibrium rather than a purely vascular abnormality.

Within this disease context, ferroptosis-related pathways may represent one mechanism contributing to early neurovascular injury. Experimental studies suggest that high-glucose stress can promote membrane lipid remodeling, disturb iron handling, and weaken antioxidant defenses. For example, upregulation of ACSL4 may increase the incorporation of PUFAs into membrane phospholipids, thereby expanding the pool of peroxidizable substrates. Persistent oxidative stress may also be associated with heme oxygenase 1 (HMOX1) induction and increased release of redox-active iron, which could favor LIP expansion under diabetic conditions (75–77). Taken together, these changes support the possibility that ferroptosis contributes to DR-associated tissue injury; however, the relative importance of this pathway, and its causal hierarchy compared with other forms of oxidative or inflammatory cell injury, remains to be defined more clearly.

Importantly, ferroptosis in DR should not be framed as a neuron-only process. Müller glia, which are central to retinal metabolic support, glutamate homeostasis, and inflammatory regulation, have also been reported to exhibit ferroptosis-related alterations under high-glucose conditions, including reduced GPX4-associated defense, altered lipid metabolism, and increased oxidative injury. Dysfunction of these glial cells may in turn amplify neuronal vulnerability and exacerbate local inflammatory and metabolic stress (75, 78, 79). Therefore, in DR, ferroptosis is more appropriately considered within a broader neuro-glial-microvascular context, with retinal ganglion cell injury representing one important component rather than the sole pathological focus.

These findings support ferroptosis as one possible link between metabolic overload and neurovascular dysfunction. The main limitation is that many observations still derive from high-glucose models, bioinformatic screening, or oxidative-stress-associated markers. Thus, in DR, ferroptosis should be framed as a candidate amplifier of metabolic and microvascular injury, with particular relevance to early neurovascular vulnerability, rather than as a single dominant pathway explaining all diabetic retinal phenotypes.

### 3.3 Optic neuritis and demyelinating neuropathies

The clinical management of ON and related demyelinating optic neuropathies remains limited by the dissociation between acute inflammatory control and long-term neuroaxonal preservation. High-dose systemic corticosteroids can accelerate visual recovery during the acute phase, but they do not consistently improve final visual outcomes or fully prevent subsequent optic atrophy and axonal degeneration (80, 81). This pattern suggests that tissue injury may continue after the initial inflammatory surge has been attenuated (82). In this setting, ferroptosis is relevant as a candidate mechanism of post-inflammatory secondary degeneration, particularly when demyelination, redox imbalance, and axon-glia metabolic uncoupling persist.

The primary pathological background of ON-related demyelinating injury includes immune-cell infiltration, activation of microglia and macrophages, oligodendrocyte damage, myelin breakdown, and impaired metabolic support for axons (83). These events differ from pressure-related RGC injury in glaucoma and chronic metabolic neurovascular dysfunction in DR. In this context, the ferroptosis-relevant issue centers on whether demyelination and inflammatory oxidative stress create local conditions that favor iron-dependent lipid injury within the optic nerve microenvironment.

Two mechanisms provide the main rationale for linking ferroptosis to demyelinating optic nerve injury. First, oligodendrocytes and myelin contain abundant iron and lipid components, and demyelination may increase local availability of redox-active iron and PUFA-rich membrane substrates (11, 52–55, 84). Second, inflammatory cytokine signaling may weaken antioxidant buffering. Experimental evidence suggests that interferon gamma (IFN-*γ*)-associated signaling can suppress System Xc⁻-related defenses, restrict cystine uptake, and reduce glutathione availability, thereby compromising GPX4-dependent lipid peroxide clearance (85). These mechanisms support a plausible route by which immune-demyelinating injury may be followed by persistent lipid peroxidation and glial-axonal stress.

Accordingly, ferroptosis-related injury in ON should be discussed at the glial-axonal interface. Oligodendrocyte injury, myelin loss, inflammatory cell activation, and impaired axon-glia metabolic coupling may jointly sustain iron accumulation, lipid peroxidation, and deficient antioxidant defense. This framing keeps the discussion close to the disease biology of demyelinating optic neuropathies and avoids overextending evidence derived from retinal RGC-centered models.

At the current stage, the translational implication should remain limited. Ferroptosis-related targets may complement immune control by limiting secondary iron- and lipid-dependent injury, but direct support in ON-specific ocular models remains insufficient. Future studies need to confirm cell-type-specific ferroptosis in oligodendrocytes, axons, and inflammatory cells, define its temporal relationship with demyelination and remyelination, and determine whether ferroptosis modulation improves visual pathway structure and function. Therefore, this section supports the mechanistic rationale for secondary neuroprotection without anticipating the delivery, long-acting modulation, and biomarker arguments developed in later sections.

These disease-specific ferroptosis-relevant patterns and their translational implications are summarized in Table 2.

**Table 2:** Disease-specific patterns through which ferroptosis-related mechanisms may contribute to representative retinal neurodegenerative diseases and optic neuropathies.

Disease model	Main clinical limitation	Dominant upstream drivers	Putative ferroptosis-relevant mechanisms	Translational implication
Glaucoma	In some patients, IOP lowering does not fully prevent progressive RGC loss	Mechanical stress, local ischemia/reperfusion, and excitotoxic stress	Axonal transport disruption, mitochondrial stress, iron dyshomeostasis, and impaired antioxidant buffering may collectively increase ferroptosis susceptibility	Combine conventional IOP control with adjunctive local neuroprotection targeting iron handling, lipid peroxidation, and antioxidant defense
Diabetic retinopathy (DR)	Anti-VEGF therapy and glycemic control mainly address late vascular manifestations and may not fully prevent early neurovascular injury	Chronic hyperglycemia, metabolic overload, and persistent oxidative/inflammatory stress	Lipid remodeling, altered iron handling, and weakened antioxidant defenses within the neurovascular unit may contribute to ferroptosis-related injury	Prioritize early neurovascular protection and identify metabolically vulnerable patients or disease stages before overt vascular progression
Inflammatory and demyelinating optic neuropathy	Acute anti-inflammatory therapy does not fully prevent later axonal degeneration and optic atrophy	Immune inflammation, demyelination, and oxidative stress	Myelin and oligodendrocyte injury may increase local iron and lipid substrate availability, while inflammatory signaling may impair System Xc⁻/GSH/GPX4-associated defense	Integrate immune control with metabolic protection aimed at limiting secondary degeneration and preserving axon–glia integrity

This table summarizes disease-specific contexts in which ferroptosis-related mechanisms may contribute to retinal or optic nerve degeneration. The evidence strength, dominant cell types, and temporal role of ferroptosis vary across disease models. Therefore, the table is intended to support comparative interpretation rather than to imply that ferroptosis has equivalent causal weight in all listed diseases.

## 4 Translational barriers to anti-ferroptotic therapies and the development of novel delivery systems

The preceding chapters have discussed the pathophysiological significance of ferroptosis in retinal neurodegenerative diseases and optic neuropathies, delineating both shared mechanisms and disease-specific contexts. However, in ophthalmic translational medicine, mechanistic targetability does not automatically equate to clinical applicability. Whether they are iron chelators, radical-trapping antioxidants, or molecular interventions targeting nodes such as GPX4 or FSP1, these agents must overcome three critical barriers to effectively penetrate the inner retina and optic nerve tissue and exert sustained neuroprotection: ocular anatomical barriers, local pharmacokinetic limitations, and safety constraints associated with long-term administration.

For retinal neurodegenerative diseases and optic neuropathies, these limitations are particularly pronounced. First, RGCs and their axons are situated behind the BRB and the vitreoretinal interface; consequently, systemic administration struggles to achieve stable, effective, and cell-specific drug exposure within the target lesions. Second, many anti-ferroptotic molecules inherently exhibit high hydrophobicity, short half-lives, or insufficient tissue selectivity, properties that are fundamentally incompatible with the hydrophilic intraocular environment and the requirement for chronic dosing in progressive diseases. Third, the therapeutic target of ferroptosis intervention is the localized dysregulation of iron homeostasis and membrane lipid peroxidation, rather than systemic iron overload. Therefore, any intervention lacking spatial selectivity carries significant risk of off-target effects.

Consequently, the core focus of this chapter is not to reiterate the mechanistic efficacy of anti-ferroptotic molecules, but rather to elucidate why these strategies still face substantial hurdles in achieving viable local neuroprotection in ophthalmic settings. Building on this premise, this chapter will examine the pharmacokinetic and anatomical obstacles encountered during intraocular application of existing molecules. Furthermore, it will explore why leveraging cross-barrier delivery systems and long-acting expression strategies is imperative for achieving effective, sustainable, and relatively selective local interventions within the target lesions.

### 4.1 Pharmacokinetic limitations

Currently, the majority of anti-ferroptotic strategies remain predicated on conventional small molecules, encompassing iron chelators, radical-trapping antioxidants, and pharmacological inhibitors designed around lipid peroxidation and antioxidant defense nodes. While these molecules frequently demonstrate definitive activity *in vitro* or within acute injury models, the primary constraint on their translational application lies not in their inherent “efficacy,” but rather in their capacity to reach and operate within the intraocular focal areas at an appropriate concentration, duration, and distribution (86, 87).

Radical-trapping antioxidants, exemplified by Ferrostatin-1 and Liproxstatin-1, fundamentally require intercalation into the lipid bilayer to intercept lipid peroxyl radicals and halt chain peroxidation reactions. This mechanism of action dictates their generally strong lipophilicity; however, it is precisely this physicochemical property that introduces a pronounced contradiction in ocular drug delivery. The vitreous cavity presents a highly hydrophilic, gel-like environment; upon entry, the diffusion of hydrophobic small molecules is restricted, and a proportion of these molecules may aggregate, thereby reducing their actual bioavailability within the inner retina. Concurrently, the internal limiting membrane (ILM) and associated reticular structures impose additional resistance to the further penetration of drug molecules into the inner retina, preventing the establishment of sufficient neuroprotective effects at the tissue level (86–89).

Although conventional iron chelators can directly attenuate the labile iron burden, their limitation lies in a pronounced mismatch between tissue selectivity and their sphere of action. Chelators with higher hydrophilicity, such as deferoxamine (DFO), exhibit limited cell membrane permeability, making it difficult for them to efficiently enter target cells within the eye to neutralize the intracellular LIP. Conversely, relatively more tissue-penetrable small molecules, such as deferiprone (DFP), while demonstrating certain retinoprotective effects in animal models, do not confine their activity solely to the focal lesions (90, 91). For retinal neurodegenerative diseases and optic neuropathies, the true target is the localized, abnormally expanded LIP, rather than systemic iron overload. Consequently, systemic chelation lacking spatial selectivity may not only interfere with the physiological iron utilization of normal neural tissue but also present a narrow therapeutic window and associated risks with long-term administration.

Retinal neurodegenerative diseases and optic neuropathies typically exhibit a chronic, progressively advancing course, indicating that anti-ferroptotic interventions cannot merely be transiently effective; they require sustained stability over extended temporal scales. However, most conventional small molecules possess limited intraocular residence times. Repeated administration introduces additional compliance burdens and operation-related risks, and may even precipitate off-target effects. Therefore, for conditions necessitating long-term maintenance of local antioxidant capacity and iron homeostasis regulation, this short-acting, fluctuating mode of drug delivery is misaligned with the chronic nature of the underlying pathological processes. Furthermore, continuous long-term administration of non-selective chelators or potent antioxidants may disrupt the normal cellular requirements for basal ROS signaling and physiological iron metabolism, thereby precipitating novel metabolic imbalances. Thus, the therapeutic goal should be normalization of pathological iron and redox states rather than their sustained global suppression.

Consequently, the core contradiction in translating anti-ferroptotic small molecules to ophthalmology is not merely whether the drug can inhibit ferroptosis, but whether it can stably reach the specific cells and subcellular compartments requiring protection without excessively perturbing normal metabolism. For this very reason, conventional free drugs frequently fail to accomplish this task independently. The subsequent strategies developed around cross-barrier delivery, microenvironment-responsive release, and long-term gene reprogramming represent more than mere technological upgrades; they constitute an essential paradigm shift required for the clinical realization of ophthalmic anti-ferroptotic therapies.

### 4.2 Active targeting and nanocarrier systems

For ferroptosis-targeted therapy, the central delivery challenge is not simply whether a drug can be administered intraocularly, but whether it can reach the relevant retinal compartment, enter the appropriate cell population, and achieve sustained activity against iron dyshomeostasis, lipid peroxidation, or antioxidant collapse. A more useful way to evaluate emerging delivery systems is therefore to classify them by platform type, representative ferroptosis-relevant cargo or target, ocular compartment reached, and current level of validation. From this perspective, the translational value of nanocarriers lies less in material novelty *per se* than in their ability to match delivery behavior with the spatial and biochemical constraints of the retinal microenvironment.

Representative emerging ocular approaches include intravitreally administered lipid nanoparticles capable of accessing inner retinal cells, biomimetic nanoparticles designed to modulate the inflammatory retinal microenvironment, and microenvironment-responsive supramolecular systems combining iron chelation with additional protective functions. For example, a deferoxamine-containing supramolecular nanoparticle has been evaluated in retinal ischemia/reperfusion injury to simultaneously target ferroptosis-related iron stress, inflammation, and oxidative injury, illustrating how delivery design may be coupled directly to ferroptosis-relevant mechanisms.

A first platform class comprises receptor-mediated and transcytosis-enabled carriers, which are designed to improve access to the inner retina. Representative strategies include arginyl-glycyl-aspartic acid (RGD)-modified nanoparticles that promote adhesion and internalization at the retinal ganglion cell surface, transferrin receptor-directed systems that exploit iron-transport-associated pathways, and intravitreally delivered lipid nanoparticles capable of traversing the internal limiting membrane and transfecting inner retinal cells such as Müller glia (92, 93). In ferroptosis-oriented applications, these systems are most relevant for delivering antioxidant or iron-regulatory cargo, or for enabling nucleic acid-based modulation of pathways such as GPX4-associated defense. The main ocular compartments reached by this class are the vitreoretinal interface and inner retinal layers. At present, the strongest support for this platform class lies in preclinical ocular evidence for barrier crossing and cell entry, whereas disease-model validation for ferroptosis-specific therapeutic benefit remains more limited.

A second platform class includes biomimetic or immune-evasive carriers, which are particularly relevant in inflammatory retinal lesions where nanoparticle clearance by activated microglia may reduce effective local exposure. Membrane-coated nanoparticles derived from apoptotic retinal cells exemplify this approach. Their translational rationale is not only immune evasion, but also local microenvironment modulation, including the possibility of shifting phagocytic or inflammatory responses toward a less damaging state while concentrating anti-ferroptotic cargo within the lesion (94, 95). In ferroptosis-relevant terms, this class is conceptually suited to disease settings in which oxidative injury, inflammation, and iron-related stress coexist. However, the current evidence base remains best characterized as proof-of-concept to early preclinical ocular support, with comparatively limited direct validation in ferroptosis-driven retinal disease models.

A third platform class comprises microenvironment-responsive and dual-function assemblies, which are intended to couple local sensing with selective release or combined pathway intervention. Representative examples include self-assembled formulations that pair iron chelation with anti-inflammatory payloads, such as DFO-containing systems designed to reduce redox-active iron while simultaneously attenuating inflammatory signaling (96). These platforms are particularly relevant when ferroptosis is framed not as an isolated molecular event, but as part of a broader neuro-glial-microvascular injury state. Their principal ocular target is not a single cell layer alone, but the stressed lesion microenvironment in which free iron, inflammatory mediators, and lipid peroxidation interact. From an evidence standpoint, this class currently occupies a preclinical but still relatively early translational position: the mechanistic rationale is strong, yet the extent of compartment-specific delivery and durable disease modification still requires more rigorous validation.

A fourth platform class includes catalytic redox-buffering nanomaterials, such as metal-organic or covalent organic frameworks and other redox-active carriers with enzyme-mimetic properties (97, 98). In principle, these systems may serve a dual role by carrying anti-ferroptotic cargo while also buffering ROS through superoxide dismutase- or catalase-like activity, thereby compensating for local depletion of endogenous antioxidant defenses. Related stimuli-responsive nanoparticles may also be engineered to activate upstream protective programs, such as NRF2-associated transcriptional responses, while limiting membrane lipid peroxidation. These platforms are most relevant for retinal compartments characterized by persistent oxidative burden, but their current status is best considered conceptual to early preclinical, particularly because long-term ocular biocompatibility, compartment selectivity, and true ferroptosis-specific efficacy remain incompletely defined.

Viewed together, these platform classes clarify an important translational point: the most advanced ocular evidence currently supports the feasibility of improving retinal access, inner retinal uptake, and local payload retention, whereas direct demonstration of durable ferroptosis-targeted neuroprotection remains much less mature. Accordingly, the near-term value of nanocarrier systems lies in enabling compartment-matched delivery of anti-ferroptotic strategies rather than in implying that any one platform has already emerged as a validated therapeutic solution. For ophthalmic translation, the key benchmark should be whether a given carrier can reproducibly connect a ferroptosis-relevant target, a defined retinal compartment, and a disease-relevant efficacy readout within the same experimental system.

### 4.3 AAV-mediated and non-viral reprogramming

For retinal neurodegenerative diseases and optic neuropathies, the key limitation of many anti-ferroptotic strategies is not simply whether an intervention can transiently suppress lipid peroxidation or iron-dependent injury, but whether protective activity can be sustained within the relevant retinal or optic nerve compartment over the long term. This issue is especially important in chronic disorders such as glaucoma, DR, and inflammatory optic neuropathies, in which repeated administration of short-lived agents may be poorly aligned with the prolonged course of tissue degeneration. A more useful way to evaluate long-acting approaches is therefore to distinguish gene-based platforms by delivery type, representative ferroptosis-relevant target, intended cellular or tissue compartment, and current level of validation.

The first and currently most advanced platform class is AAV-mediated gene augmentation, which is best suited for sustained reinforcement of intrinsic cellular defense programs (99). In ferroptosis-oriented applications, the most representative target is GPX4, given its central role in detoxifying phospholipid hydroperoxides. Within the eye, this strategy is particularly relevant for terminally differentiated retinal cells, including RGCs, in which durable expression may be more compatible with chronic injury states than repeated short-term pharmacological inhibition. Its principal translational advantage is sustained local expression, whereas its main unresolved issues include expression control, cell specificity, and the long-term consequences of chronically enhancing antioxidant or iron-regulatory pathways beyond the primary lesion.

Within the AAV platform, GPX4 remains one of the most compelling ferroptosis-relevant candidate genes because it occupies a central position in phospholipid peroxide detoxification. Experimental studies indicating that AAV-mediated, RGC-directed GPX4 overexpression can improve neuronal survival and regeneration-related outcomes in models of optic nerve injury and glaucoma provide the strongest support for this platform class (66). At present, however, the evidence should still be regarded as preclinical disease-model validation rather than as a clinically established therapeutic route.

A second platform class comprises non-viral gene delivery or gene-expression approaches, which are less optimized for maximal transduction efficiency but offer advantages in cargo flexibility, lower viral burden, and potentially broader upstream metabolic modulation. In the current context, the most representative example is sustained intraocular transferrin expression achieved through plasmid-based electroporation to the ciliary muscle. In ferroptosis-relevant terms, this strategy is conceptually aligned with upstream remodeling of iron handling rather than direct blockade of the execution phase of lipid peroxidation (100, 101). Current support for this platform is best characterized as proof-of-concept to preclinical ocular evidence, with encouraging biological rationale but less mature disease-specific validation than that available for AAV-GPX4-based approaches.

Across these platform classes, the major unresolved issue is not whether long-term expression can be achieved, but whether it can be achieved with sufficient localization, controllability, and physiological restraint. Continuous expression of molecules such as GPX4, FSP1, or transferrin could theoretically interfere with basal redox signaling or physiological iron utilization in non-target tissue. Thus, the central benchmark for long-acting anti-ferroptotic gene strategies should not be duration alone, but the ability to link a defined ferroptosis-relevant target, a specific retinal or optic nerve compartment, and a disease-relevant efficacy readout without imposing unacceptable metabolic perturbation.

From this perspective, AAV-based and non-viral strategies should be viewed as complementary rather than competing modalities. The former is currently stronger for stable enhancement of intrinsic cellular defense, whereas the latter may be more suitable for flexible upstream remodeling of the local metabolic environment. Together, they are relevant not simply because they achieve “gene delivery,” but because they offer a potential route for converting short-acting pharmacological inhibition into a more durable state of compartment-matched metabolic protection.

## 5 Biomarker-guided translation of ferroptosis-targeted neuroprotection

For retinal neurodegenerative diseases and optic neuropathies, overcoming delivery barriers does not signify the completion of the translational pathway. As research advances toward clinical application, the fundamental question shifts from whether ferroptosis can be targeted to which intervention layer should be prioritized, within which specific disease contexts and pathological stages, and how reproducible metrics can be utilized to support patient stratification and therapeutic evaluation. In other words, the developmental trajectory of ophthalmic anti-ferroptotic therapies must evolve beyond merely engaging a molecular target; it must encompass the refinement of comprehensive strategies for disease assessment and patient stratification.

Guided by this paradigm, this chapter will first outline the hierarchy of anti-ferroptotic interventions that currently possess the highest translational potential from a clinical decision-making perspective. These encompass iron homeostasis remodeling, lipid peroxidation blockade, endogenous antioxidant defense enhancement, mitochondrial metabolic buffering, and long-acting gene reprogramming. Subsequently, it will examine the critical role of AI-assisted multi-omics and multimodal biomarker frameworks in patient stratification, the identification of optimal intervention windows, and the longitudinal monitoring of therapeutic efficacy.

### 5.1 Prioritizing anti-ferroptotic intervention layers by disease context and stage

Interventions targeting ferroptosis should not be understood as blockade of a single molecular node, but rather as mechanistically distinct and potentially complementary layers of intervention. Based on current evidence, anti-ferroptotic strategies can be broadly grouped into three categories: limiting reactive iron accumulation, interrupting membrane lipid peroxidation or reinforcing endogenous antioxidant defense, and buffering mitochondria-associated metabolic stress. These intervention layers correspond to different positions along the ferroptosis cascade, and their relevance is likely to vary according to disease context, pathological stage, and the dominant form of microenvironmental stress.

Iron-homeostasis remodeling represents an intuitive entry point because it aims to restrict pathological expansion of the LIP and thereby attenuate iron-driven oxidative injury (102). DFO and DFP have provided early proof-of-concept support in ophthalmic or iron-overload-associated retinal injury settings (90, 91, 103). However, retinal neurodegenerative diseases and optic neuropathies are not systemic iron overload disorders. Their pathological basis is more plausibly characterized by local intracellular iron redistribution and expansion of redox-active iron pools. Therefore, the translational goal is not simple iron depletion, but selective control of pathological iron mobilization within disease-relevant compartments. In this regard, upstream regulation of ferritinophagy, particularly through NCOA4-related mechanisms, may offer a more targeted route for limiting stress-induced iron release (32, 104–106). At present, iron-directed intervention should still be regarded as context-dependent and most informative in disease settings where catalytic iron dysregulation is prominent.

Compared with iron-directed approaches, lipid-peroxidation blockade acts closer to the execution phase of ferroptosis. Ferrostatin-1 and Liproxstatin-1 can interrupt lipid peroxyl chain reactions and have shown protective effects in experimental optic neuropathy or glaucoma-related models (39, 107, 108). For chronic disease states, however, execution-phase blockade alone may be insufficient if persistent metabolic stress, glial dysregulation, or iron dyshomeostasis continuously regenerates oxidative pressure. This has led to increasing interest in reinforcing endogenous protective systems, including preservation of the GSH/GPX4 axis, activation of NRF2-related defense pathways, and support of FSP1-CoQ10-associated buffering (5, 7, 40, 109). Molecules such as nicotinamide or CoQ10 may also support broader antioxidant resilience (110–112). This intervention layer may therefore be most relevant when lipid peroxidation and antioxidant-defense failure are prominent, but sustained amplification of antioxidant programs still requires caution because basal redox signaling and physiological metabolic regulation may also be affected.

Mitochondrial buffering may serve as an upstream supportive layer in disease contexts dominated by sustained metabolic stress, including DR, ischemia-reperfusion injury, and selected mitochondria-associated optic neuropathies. Mitochondria-targeted antioxidants such as mitoquinone (MitoQ) and mitochondria-targeted TEMPO (MitoTEMPO), together with strategies modulating mitophagy or mitochondrial quality control, may reduce mitochondrial ROS generation and thereby limit downstream membrane lipid damage (39, 113–115). Nevertheless, this layer should be framed as complementary rather than substitutive. Its practical value depends on whether the intervention can reach the relevant cell type and subcellular compartment, and whether mitochondrial stress is truly a dominant contributor in the specific disease stage being treated.

Taken together, these intervention layers should not be interpreted as a fixed therapeutic ranking. Iron-homeostasis remodeling may be more informative when catalytic iron dysregulation is prominent; lipid-peroxidation blockade and antioxidant reinforcement may be more relevant when execution-phase injury and defense failure are evident; and mitochondrial buffering may be especially pertinent when chronic metabolic stress is a dominant feature. For retinal neurodegenerative diseases and optic neuropathies, future clinical translation is therefore more likely to depend on disease-specific and stage-specific prioritization than on identification of a single universally optimal anti-ferroptotic inhibitor.

The therapeutic window for ferroptosis-targeted neuroprotection is also likely to vary across disease stages. In relatively early disease, when RGCs and supporting retinal cells remain structurally viable but exhibit metabolic stress, iron dyshomeostasis, or impaired antioxidant defense, anti-ferroptotic intervention may have the greatest potential to preserve reversible cellular dysfunction before substantial neuronal or axonal loss occurs. During progressive disease, ferroptosis-targeted therapy may remain useful as an adjunct to conventional treatment if ongoing lipid peroxidation and redox imbalance continue to contribute to secondary degeneration. By contrast, once extensive RGC loss, axonal degeneration, or tissue remodeling has become established, inhibition of ferroptotic injury alone is unlikely to restore irreversibly lost neural structures, although it may still protect residual vulnerable cells. Accordingly, the optimal therapeutic window is likely to precede advanced structural loss and should be defined using disease-specific molecular, structural, and functional biomarkers rather than chronological disease duration alone.

### 5.2 Refining patient stratification and therapeutic windows through biomarker frameworks

The clinical translation of ferroptosis-targeted neuroprotection will depend not only on target selection and delivery feasibility, but also on whether patients and disease stages can be stratified according to ferroptosis-relevant vulnerability. This requirement is particularly important for retinal neurodegenerative diseases and optic neuropathies, because ferroptosis is unlikely to act uniformly across glaucoma, DR, and inflammatory or demyelinating optic neuropathies. Accordingly, the main value of biomarker development is not simply to detect retinal or optic nerve damage, but to determine whether iron dyshomeostasis, lipid peroxidation, and antioxidant-defense failure are likely to contribute to a modifiable injury state.

Current structural and functional assessments, including optical coherence tomography (OCT), visual field testing, and electrophysiological measures, remain indispensable for documenting tissue damage and functional decline. Recent glaucoma imaging guidelines and automated image-analysis studies further illustrate how standardized annotation, quality control, and deep learning can improve reproducible extraction of conventional structural phenotypes from fundus photography and OCT (116, 117). However, such structural outputs remain markers of disease phenotype rather than direct evidence of ferroptosis-related pathway activity. Similarly, MDA, 4-HNE, GSH, and related markers in ocular fluids or blood may provide indirect information on oxidative stress and lipid peroxidation burden, but they remain insufficiently specific to distinguish ferroptosis from broader redox injury (110, 118–120). Thus, the major limitation is not the absence of measurable signals, but the lack of an integrated framework that can connect molecular stress, tissue-level structural change, and functional outcomes in a clinically interpretable manner.

Within this context, AI-assisted multi-omics may help identify candidate ferroptosis-related signatures involving iron handling, lipid metabolism, antioxidant defense, and inflammatory signaling networks. Consistent with recent neuro-ophthalmic AI consensus recommendations, the development of such models will require multimodal data integration together with rigorous attention to data quality, interpretability, external validation, and population-level generalizability (121). Compared with single-marker approaches, this strategy is better suited to capturing coordinated pathway-level remodeling within the retinal microenvironment. In DR, for example, bioinformatic analyses involving HMOX1, prostaglandin-endoperoxide synthase 2 (PTGS2), weighted gene co-expression network analysis, and machine-learning refinement may help narrow the candidate space and generate more testable ferroptosis-relevant profiles (75–77). Nevertheless, these signatures should not be treated as clinically validated biomarkers. Their current role is better defined as hypothesis generation, candidate prioritization, and retrospective stratification, because robustness across cohorts, tissues, disease stages, and analytical platforms remains insufficient.

A more clinically useful strategy is therefore unlikely to rely on a single molecule, imaging parameter, or omics-derived signature. Because ferroptosis is dynamic and microenvironment-dependent, candidate markers may indicate susceptibility, association, or downstream consequence rather than ongoing pathway execution. Multimodal integration may provide a more feasible framework by combining fluid-based redox markers, iron-related imaging such as quantitative susceptibility mapping (QSM), activatable probes targeting Fe^2^⁺ or lipid peroxidation, and omics-derived signatures (122–124). This direction is consistent with the broader evolution of ophthalmic imaging toward quantitative, multimodal, and AI-assisted integration for disease characterization and clinical decision support (125). These modalities remain at uneven levels of maturity, and most still provide supportive rather than definitive evidence of active ferroptosis *in vivo*. However, when interpreted together, they may help approximate whether a lesion is iron-dominant, lipid-peroxidation–dominant, antioxidant-defense–deficient, or primarily driven by broader metabolic or inflammatory stress.

Ultimately, a clinically useful ferroptosis biomarker framework should function as an integrated decision-support system rather than as a single-test solution. Its purpose should be to support three linked decisions: whether a patient exhibits ferroptosis-relevant vulnerability, whether the disease remains within a potentially modifiable therapeutic window, and whether an intervention alters molecular profiles in parallel with structural or functional outcomes. Until such cross-validated systems are established, biomarker research should be regarded as an enabling translational frontier rather than a mature clinical toolkit.

An integrated roadmap linking disease context, delivery barriers, intervention layers, and biomarker-guided implementation is shown in Figure 3, and the major biomarker domains, representative readouts, translational uses, and current limitations are summarized in Table 3.

**Figure 3:**
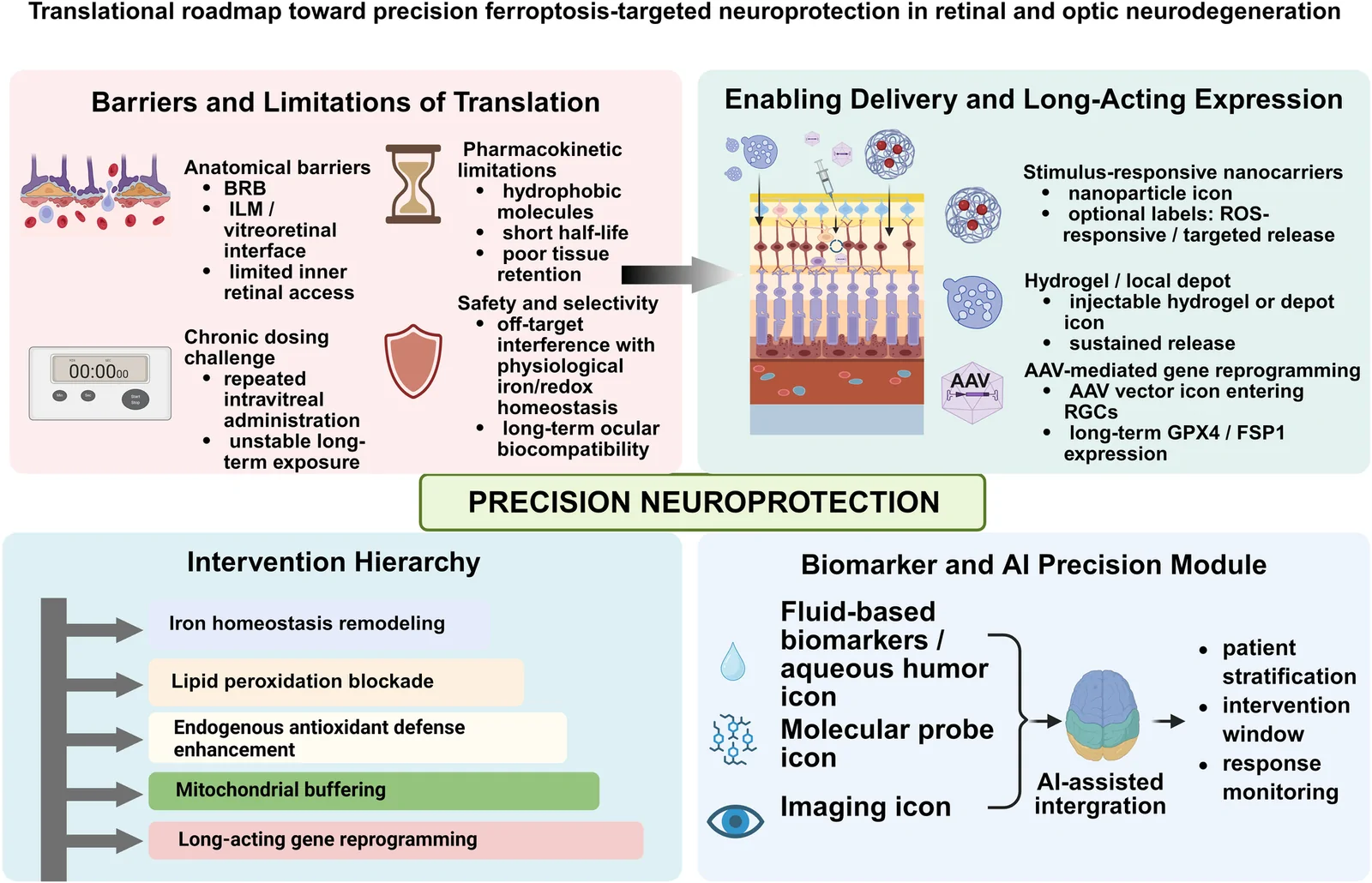
Translational roadmap toward precision ferroptosis-targeted neuroprotection in retinal neurodegenerative diseases and optic neuropathies. Mechanical/ischemic, metabolic, and immune-inflammatory stress represent three major upstream contexts in glaucoma, DR, and inflammatory/demyelinating optic neuropathies. Shared translational barriers include limited retinal access, insufficient local exposure, repeated dosing burden, and safety concerns. Potential intervention layers include iron regulation, lipid peroxidation blockade, antioxidant reinforcement, mitochondrial buffering, and long-acting gene reprogramming. Delivery platforms and biomarker frameworks may support translation, but current validation remains uneven. Created with http://biorender.com

**Table 3:** Translational biomarker-support framework for ferroptosis-related stratification and monitoring in retinal and optic nerve neurodegeneration.

Biomarker domain	Representative readouts or approaches	What it may reflect	Main translational use	Current evidence status	Main limitation
Fluid-based oxidative/redox markers	MDA, 4-HNE, GSH and related oxidative stress products in aqueous humor, vitreous, or blood	General oxidative stress, lipid peroxidation burden, altered redox state	Preliminary risk enrichment and longitudinal adjunct monitoring	Widely measurable but currently limited to indirect supportive evidence	Poor ferroptosis specificity; influenced by systemic and non-ferroptotic oxidative injury
Iron-related imaging readouts	Susceptibility-based magnetic resonance imaging (MRI) readouts, including quantitative susceptibility mapping (QSM)	Iron accumulation or redistribution in relevant tissue compartments	Identification of iron-dominant disease contexts and support for patient stratification	Promising but still limited for ophthalmic ferroptosis applications	Limited disease specificity; does not by itself confirm active ferroptotic injury
Activatable molecular probes	Fe^2^⁺-responsive probes, lipid peroxidation-sensitive probes, near-infrared (NIR)/Förster resonance energy transfer (FRET)-based dynamic reporters	Labile iron, lipid peroxide formation, or related microenvironmental shifts	Mechanistic interrogation and potential dynamic monitoring of ferroptosis-relevant states	Conceptual to preclinical	Limited clinical applicability; incomplete standardization and tissue validation
Multi-omics candidate signatures	Transcriptomic, metabolomic, and network-level signatures involving iron handling, lipid metabolism, antioxidant defense, and inflammatory pathways	Ferroptosis-relevant susceptibility profiles rather than direct pathway execution	Candidate discovery, disease subtyping, and hypothesis generation	Exploratory to retrospective preclinical/clinical bioinformatic support	Cohort dependence; platform variability; limited cross-stage and cross-tissue validation
AI-assisted integrative models	Machine learning–based integration of omics, imaging, and fluid biomarkers	Composite ferroptosis-relevant risk patterns and latent disease subgroups	Patient stratification, therapeutic window estimation, and response modeling	Early translational and not yet clinically validated	Model generalizability, interpretability, and prospective validation remain insufficient
Multimodal integrated monitoring	Combined use of fluid markers, imaging, probes, and omics-derived signatures	A more comprehensive approximation of ferroptosis-related vulnerability and treatment responsiveness	Potentially comprehensive framework for stratification, window identification, and longitudinal monitoring	Strategically attractive but still immature	Requires cross-platform standardization, reproducibility, and disease-relevant validation

This table summarizes biomarker domains according to their likely translational roles in ferroptosis-related patient stratification and monitoring. The wording is intentionally cautious because most current readouts reflect susceptibility or associated stress states rather than definitive proof of active ferroptotic injury *in vivo*.

## 6 Discussion and future directions

Ferroptosis provides a useful framework for interpreting how iron dyshomeostasis, phospholipid peroxidation, and insufficient antioxidant defense may connect heterogeneous retinal and optic nerve insults with sustained neurodegenerative injury. In the retinal neurovascular unit and optic nerve axis, this process should not be viewed as exclusively neuron-autonomous; rather, RGC vulnerability is shaped by microglial activation, Müller glia and astrocyte dysfunction, BRB disruption, oligodendrocyte injury, and impaired axon–glia metabolic coupling. This network-level interpretation is consistent with the disease-specific differences discussed in glaucoma, DR, and inflammatory or demyelinating optic neuropathies. At the same time, the current evidence remains uneven. Therefore, future work should move beyond documenting ferroptosis-related marker changes and clarify when ferroptosis acts as a causal driver, an execution-phase mechanism, or an associated stress response. This would require cell-specific ferroptosis readouts, disease-relevant structural and functional endpoints, compartment-matched delivery systems, and biomarker frameworks capable of linking molecular vulnerability with disease stage and therapeutic response.

Importantly, the current preclinical evidence should be interpreted within a broader regulated cell-death framework rather than assuming that ferroptosis acts as an isolated or exclusive execution pathway. Apoptosis has long been implicated in RGC degeneration, while RIPK1/RIPK3/MLKL-dependent necroptotic signaling has also been demonstrated in experimental models of excitotoxic, ischemic, and optic nerve injury. These pathways share several upstream stressors with ferroptosis, including mitochondrial dysfunction, ROS accumulation, glutamate excitotoxicity, and inflammatory signaling, and may therefore coexist or predominate differently across disease stages and retinal cell populations. Within this broader context, the strength of ferroptosis-specific evidence remains uneven. Experimental glaucoma and optic nerve injury currently provide comparatively stronger support, with studies demonstrating iron dyshomeostasis, ferritinophagy, lipid peroxidation, and impaired GPX4-associated defense, together with RGC protection following pharmacological ferroptosis inhibition. GLAST-deficient models further link impaired glutamate handling to ferroptosis-related RGC loss, while RGC-directed AAV-mediated GPX4 overexpression provides complementary genetic evidence for neuroprotection and regenerative benefit. By contrast, evidence in diabetic retinopathy remains more dependent on high-glucose cellular models and ferroptosis-associated changes in glial or microvascular compartments, whereas pharmacological iron modulation in demyelinating optic nerve injury provides supportive but less pathway-specific evidence. Accordingly, pharmacological rescue or changes in iron accumulation, GPX4, and lipid peroxidation alone should not be regarded as definitive proof that ferroptosis is the exclusive mode of RGC death. Stronger causal validation will require convergence of ferroptosis-specific biochemical readouts, genetic perturbation, disease-relevant structural and functional outcomes, and, where appropriate, parallel assessment of apoptotic and necroptotic pathways (126, 127).

Overall, ferroptosis-targeted neuroprotection is best regarded as a disease- and stage-dependent translational framework rather than a universal therapeutic solution for retinal neurodegeneration and optic neuropathies. Its clinical value will depend on stronger causal validation, safer and more selective intraocular delivery, and biomarker-guided patient stratification. With these advances, anti-ferroptotic strategies may eventually complement existing treatments by addressing secondary neurovascular and axon–glia injury that can persist beyond control of the primary disease insult.

## Data Availability

No new data were created or analyzed in this study. Data sharing is not applicable to this article.

## References

[1] Zhu J Chen H Wu J Li S Lin W Wang N. Ferroptosis in glaucoma: a promising avenue for therapy. Adv Biol (Weinh). (2024) 8:e2300530. 10.1002/adbi.202300530 38411382

[2] Kagan VE Mao G Qu F Angeli JPF Doll S Croix CS. Oxidized arachidonic and adrenic PEs navigate cells to ferroptosis. Nat Chem Biol. (2017) 13:81–90. 10.1038/nchembio.2238 27842066 PMC5506843

[3] Baltan S Inman DM Danilov CA Morrison RS Calkins DJ Horner PJ. Metabolic vulnerability disposes retinal ganglion cell axons to dysfunction in a model of glaucomatous degeneration. J Neurosci. (2010) 30:5644–52. 10.1523/JNEUROSCI.5956-09.2010 20410117 PMC2884009

[4] Dixon SJ Stockwell BR. The role of iron and reactive oxygen species in cell death. Nat Chem Biol. (2014) 10:9–17. 10.1038/nchembio.1416 24346035

[5] Wolpaw AJ Shimada K Skouta R Welsch ME Akavia UD Pe'er D. Modulatory profiling identifies mechanisms of small molecule-induced cell death. Proc Natl Acad Sci U S A. (2011) 108:E771–780. 10.1073/pnas.1106149108 21896738 PMC3182736

[6] Bridges RJ Natale NR Patel SA. System xc^−^ cystine/glutamate antiporter: an update on molecular pharmacology and roles within the CNS. Br J Pharmacol. (2012) 165:20–34. 10.1111/j.1476-5381.2011.01480.x 21564084 PMC3252963

[7] Song X Long D. Nrf2 and ferroptosis: a new research direction for neurodegenerative diseases. Front Neurosci. (2020) 14:267. 10.3389/fnins.2020.00267 32372896 PMC7186402

[8] Son JL Soto I Oglesby E Lopez-Roca T Pease ME Quigley HA. Glaucomatous optic nerve injury involves early astrocyte reactivity and late oligodendrocyte loss. Glia. (2010) 58:780–9. 10.1002/glia.20962 20091782

[9] Guo X Wei R Yin X Yang G. Crosstalk between neuroinflammation and ferroptosis: implications for Parkinson's Disease progression. Front Pharmacol. (2025) 16:1528538. 10.3389/fphar.2025.1528538 40183096 PMC11966490

[10] Au NPB Ma CHE. Neuroinflammation, microglia and implications for retinal ganglion cell survival and axon regeneration in traumatic optic neuropathy. Front Immunol. (2022) 13:860070. 10.3389/fimmu.2022.860070 35309305 PMC8931466

[11] Hazell AS Rao KV Danbolt NC Pow DV Butterworth RF. Selective down-regulation of the astrocyte glutamate transporters GLT-1 and GLAST within the medial thalamus in experimental wernicke's Encephalopathy. J Neurochem. (2001) 78:560–8. 10.1046/j.1471-4159.2001.00436.x 11483659

[12] Nnah IC Wessling-Resnick M. Brain iron homeostasis: a focus on microglial iron. Pharmaceuticals (Basel). (2018) 11:129. 10.3390/ph11040129 30477086 PMC6316365

[13] Pajarillo E Rizor A Lee J Aschner M Lee E. The role of astrocytic glutamate transporters GLT-1 and GLAST in neurological disorders: potential targets for neurotherapeutics. Neuropharmacology. (2019) 161:107559. 10.1016/j.neuropharm.2019.03.002 30851309 PMC6731169

[14] Ulhaq ZS Bittencourt GB Soraya GV Istifiani LA Pamungkas SA Ogino Y. Association between glaucoma susceptibility with combined defects in mitochondrial oxidative phosphorylation and fatty acid beta oxidation. Mol Aspects Med. (2024) 96:101238. 10.1016/j.mam.2023.101238 38215610

[15] Yu DY Cringle SJ Balaratnasingam C Morgan WH Yu PK Su EN. Retinal ganglion cells: energetics, compartmentation, axonal transport, cytoskeletons and vulnerability. Prog Retin Eye Res. (2013) 36:217–46. 10.1016/j.preteyeres.2013.07.001 23891817

[16] Pope LE Dixon SJ. Regulation of ferroptosis by lipid metabolism. Trends Cell Biol. (2023) 33:1077–87. 10.1016/j.tcb.2023.05.003 37407304 PMC10733748

[17] Gabrielle PH. Lipid metabolism and retinal diseases. Acta Ophthalmol. (2022) 100(Suppl 269):3–43. 10.1111/aos.15226 36117363

[18] Liang D Minikes AM Jiang X. Ferroptosis at the intersection of lipid metabolism and cellular signaling. Mol Cell. (2022) 82:2215–27. 10.1016/j.molcel.2022.03.022 35390277 PMC9233073

[19] Goldsteins G Hakosalo V Jaronen M Keuters MH Lehtonen Š Koistinaho J. CNS Redox homeostasis and dysfunction in neurodegenerative diseases. Antioxidants (Basel). (2022) 11:405. 10.3390/antiox11020405 35204286 PMC8869494

[20] Yan N Zhang J. Iron metabolism, ferroptosis, and the links with Alzheimer's Disease. Front Neurosci. (2019) 13:1443. 10.3389/fnins.2019.01443 32063824 PMC7000453

[21] Huang Q Zhang H Chen S Wang Y Zhou J. Ferroptosis in central nervous system injuries: molecular mechanisms, diagnostic approaches, and therapeutic strategies. Front Cell Neurosci. (2025) 19:1593963. 10.3389/fncel.2025.1593963 40766185 PMC12321900

[22] Youale J Bigot K Kodati B Jaworski T Fan Y Nsiah NY. Neuroprotective effects of transferrin in experimental glaucoma models. Int J Mol Sci. (2022) 23:12753. 10.3390/ijms232112753 36361544 PMC9659282

[23] Gao M Yi J Zhu J Minikes AM Monian P Thompson CB. Role of mitochondria in ferroptosis. Mol Cell. (2019) 73:354–363.e3. 10.1016/j.molcel.2018.10.042 30581146 PMC6338496

[24] Liang D Jiang X. START Smuggling CoQ to fight ferroptosis. Nat Cell Biol. (2023) 25:207–8. 10.1038/s41556-022-01044-1 36658221

[25] Laha B Stafford BK Huberman AD. Regenerating optic pathways from the eye to the brain. Science. (2017) 356:1031–4. 10.1126/science.aal5060 28596336 PMC6333302

[26] Díaz-Coránguez M Ramos C Antonetti DA. The inner blood-retinal barrier: cellular basis and development. Vision Res. (2017) 139:123–37. 10.1016/j.visres.2017.05.009 PMC5723228 28619516

[27] Sterling J Guttha S Song Y Song D Hadziahmetovic M Dunaief JL. Iron importers Zip8 and Zip14 are expressed in retina and regulated by retinal iron levels. Exp Eye Res. (2017) 155:15–23. 10.1016/j.exer.2016.12.008 28057442 PMC5359041

[28] Liu Q Liu CQ Yi WZ Ouyang PW Yang BF Liu Q. Ferroptosis contributes to microvascular dysfunction in diabetic retinopathy. Am J Pathol. (2024) 194:1078–89. 10.1016/j.ajpath.2024.01.019 38417697

[29] Huang H. Pericyte-endothelial interactions in the retinal microvasculature. Int J Mol Sci. (2020) 21:7413. 10.3390/ijms21197413 33049983 PMC7582747

[30] Wu H Liu Q Shan X Gao W Chen Q. ATM Orchestrates ferritinophagy and ferroptosis by phosphorylating NCOA4. Autophagy. (2023) 19:2062–77. 10.1080/15548627.2023.2170960 36752571 PMC10283418

[31] Mancias JD Wang X Gygi SP Harper JW Kimmelman AC. Quantitative proteomics identifies NCOA4 as the cargo receptor mediating ferritinophagy. Nature. (2014) 509:105–9. 10.1038/nature13148 24695223 PMC4180099

[32] Yao F Peng J Zhang E Ji D Gao Z Tang Y. Pathologically high intraocular pressure disturbs normal iron homeostasis and leads to retinal ganglion cell ferroptosis in glaucoma. Cell Death Differ. (2023) 30:69–81. 10.1038/s41418-022-01046-4 35933500 PMC9883496

[33] Wu Y Song J Wang Y Wang X Culmsee C Zhu C. The potential role of ferroptosis in neonatal brain injury. Front Neurosci. (2019) 13:115. 10.3389/fnins.2019.00115 30837832 PMC6382670

[34] Bouchaoui H Mahoney-Sanchez L Garçon G Berdeaux O Alleman LY Devos D. ACSL4 And the lipoxygenases 15/15B are pivotal for ferroptosis induced by iron and PUFA dyshomeostasis in dopaminergic neurons. Free Radic Biol Med. (2023) 195:145–57. 10.1016/j.freeradbiomed.2022.12.086 36581060

[35] Liu Y Lu S Wu LL Yang L Yang L Wang J. The diversified role of mitochondria in ferroptosis in cancer. Cell Death Dis. (2023) 14:519. 10.1038/s41419-023-06045-y 37580393 PMC10425449

[36] Ye H Feng Y Xiang W Lin Z Li Y Hu W. Ferroptosis contributes to retinal ganglion cell loss in GLAST knockout mouse model of normal tension glaucoma. Invest Ophthalmol Vis Sci. (2025) 66:26. 10.1167/iovs.66.5.26 PMC12110597 40402516

[37] Fan G Liu M Liu J Huang Y. The initiator of neuroexcitotoxicity and ferroptosis in ischemic stroke: glutamate accumulation. Front Mol Neurosci. (2023) 16:1113081. 10.3389/fnmol.2023.1113081 37033381 PMC10076579

[38] Zhang JB Jia X Cao Q Chen YT Tong J Lu GD. Ferroptosis-regulated cell death as a therapeutic strategy for neurodegenerative diseases: current status and future prospects. ACS Chem Neurosci. (2023) 14:2995–3012. 10.1021/acschemneuro.3c00406 37579022

[39] Guo M Zhu Y Shi Y Meng X Dong X Zhang H. Inhibition of ferroptosis promotes retina ganglion cell survival in experimental optic neuropathies. Redox Biol. (2022) 58:102541. 10.1016/j.redox.2022.102541 36413918 PMC9679710

[40] Doll S Freitas FP Shah R Aldrovandi M Da Silva MC Ingold I. FSP1 Is a glutathione-independent ferroptosis suppressor. Nature. (2019) 575:693–8. 10.1038/s41586-019-1707-0 31634899

[41] Yan R Lin B Jin W Tang L Hu S Cai R. NRF2, A superstar of ferroptosis. Antioxidants (Basel). (2023) 12:1739. 10.3390/antiox12091739 37760042 PMC10525540

[42] Li M Chen M Li H Gao D Zhao L Zhu M. Glial cells improve Parkinson's Disease by modulating neuronal function and regulating neuronal ferroptosis. Front Cell Dev Biol. (2024) 12:1510897. 10.3389/fcell.2024.1510897 39830208 PMC11739109

[43] Li J Jia B Cheng Y Song Y Li Q Luo C. Targeting molecular mediators of ferroptosis and oxidative stress for neurological disorders. Oxid Med Cell Longevity. (2022) 2022:3999083. 10.1155/2022/3999083 PMC9337979 35910843

[44] Goshi N Lam D Bogguri C George VK Sebastian A Cadena J. Direct effects of prolonged TNF-α and IL-6 exposure on neural activity in human iPSC-derived neuron-astrocyte co-cultures. Front Cell Neurosci. (2025) 19:1512591. 10.3389/fncel.2025.1512591 40012566 PMC11860967

[45] Abdukarimov N Kokabi K Kunz J. Ferroptosis and iron homeostasis: molecular mechanisms and neurodegenerative disease implications. Antioxidants (Basel). (2025) 14:527. 10.3390/antiox14050527 40427409 PMC12108473

[46] Wang Q Liu J Zhang Y Li Z Zhao Z Jiang W. Microglial CR3 promotes neuron ferroptosis via NOX2-mediated iron deposition in rotenone-induced experimental models of Parkinson's Disease. Redox Biol. (2024) 77:103369. 10.1016/j.redox.2024.103369 39357423 PMC11471230

[47] Cui Y Zhang Z Zhou X Zhao Z Zhao R Xu X. Microglia and macrophage exhibit attenuated inflammatory response and ferroptosis resistance after RSL3 stimulation via increasing Nrf2 expression. J Neuroinflammation. (2021) 18:249. 10.1186/s12974-021-02231-x 34717678 PMC8557003

[48] Ryan SK Zelic M Han Y Teeple E Chen L Sadeghi M. Microglia ferroptosis is regulated by SEC24B and contributes to neurodegeneration. Nat Neurosci. (2023) 26:12–26. 10.1038/s41593-022-01221-3 36536241 PMC9829540

[49] Li X Chu Y Ma R Dou M Li S Song Y. Ferroptosis as a mechanism of oligodendrocyte loss and demyelination in experimental autoimmune encephalomyelitis. J Neuroimmunol. (2022) 373:577995. 10.1016/j.jneuroim.2022.577995 36327618

[50] Looser ZJ Faik Z Ravotto L Zanker HS Jung RB Werner HB. Oligodendrocyte-axon metabolic coupling is mediated by extracellular K+ and maintains axonal health. Nat Neurosci. (2024) 27:433–48. 10.1038/s41593-023-01558-3 38267524 PMC10917689

[51] Cerasuolo M Di Meo I Auriemma MC Trojsi F Maiorino MI Cirillo M. Iron and ferroptosis more than a suspect: beyond the most common mechanisms of neurodegeneration for new therapeutic approaches to cognitive decline and dementia. Int J Mol Sci. (2023) 24:9637. 10.3390/ijms24119637 37298586 PMC10253771

[52] Rayatpour A Foolad F Heibatollahi M Khajeh K Javan M. Ferroptosis inhibition by deferiprone, attenuates myelin damage and promotes neuroprotection in demyelinated optic nerve. Sci Rep. (2022) 12:19630. 10.1038/s41598-022-24152-2 36385152 PMC9668997

[53] Xie C Wu N Guo J Ma L Zhang C. The key role of the ferroptosis mechanism in neurological diseases and prospects for targeted therapy. Front Neurosci. (2025) 19:1591417. 10.3389/fnins.2025.1591417 40421132 PMC12104224

[54] Li Y Wang B Yang J Liu R Xie J Wang J. Iron overload causes ferroptosis but not apoptosis in MO3.13 oligodendrocytes. Neurochem Res. (2023) 48:830–8. 10.1007/s11064-022-03807-6 36352276

[55] Qin D Li D Wang C Guo S. Ferroptosis and central nervous system demyelinating diseases. J Neurochem. (2023) 165:759–71. 10.1111/jnc.15831 37095635

[56] Tham YC Li X Wong TY Quigley HA Aung T Cheng CY. Global prevalence of glaucoma and projections of glaucoma burden through 2040: a systematic review and meta-analysis. Ophthalmology. (2014) 121:2081–90. 10.1016/j.ophtha.2014.05.013 24974815

[57] Allison K Patel D Alabi O. Epidemiology of glaucoma: the past, present, and predictions for the future. Cureus. (2020) 12:e11686. 10.7759/cureus.11686 33391921 PMC7769798

[58] Pang IH Clark AF. Inducible rodent models of glaucoma. Prog Retin Eye Res. (2020) 75:100799. 10.1016/j.preteyeres.2019.100799 31557521 PMC7085984

[59] Miao Y Zhao GL Cheng S Wang Z Yang XL. Activation of retinal glial cells contributes to the degeneration of ganglion cells in experimental glaucoma. Prog Retin Eye Res. (2023) 93:101169. 10.1016/j.preteyeres.2023.101169 36736070

[60] Coviltir V Burcel MG Baltă G Marinescu MC. Interplay between ocular ischemia and glaucoma: an update. Int J Mol Sci. (2024) 25:12400. 10.3390/ijms252212400 39596463 PMC11594906

[61] Yang M So KF Lam WC Yin Lo AC. Ferroptosis and glaucoma: implications in retinal ganglion cell damage and optic nerve survival. Neural Regen Res. (2023) 18:545–6. 10.4103/1673-5374.350196 36018170 PMC9727424

[62] Hu X Zhao GL Xu MX Zhou H Li F Miao Y. Interplay between müller cells and microglia aggravates retinal inflammatory response in experimental glaucoma. J Neuroinflammation. (2021) 18:303. 10.1186/s12974-021-02366-x 34952606 PMC8705189

[63] Cao Y Yin X Wu L Huang D Wang Z Wu F. High-efficiency ocular delivery of brain-derived neurotrophic factor and oligomycin for neuroprotection in glaucoma. Adv Mater. (2025) 37:e2500623. 10.1002/adma.202500623 40357695

[64] Li SY Zhou H Zhao G Ding WW Zhang Y Wang YC. Tumor necrosis factor alpha-mediated interaction between microglia and müller cells exacerbates retinal ganglion cell damage in experimental glaucoma. Neurosci Bull. (2026) 42:127–52. 10.1007/s12264-025-01478-1 40884715 PMC12789370

[65] Unraveling the role of microglial activation and immune regulation in glaucomatous neurodegeneration insights into its dynamic interactions with retinal cells. Available online at: https://www.maxapress.com/article/doi/10.48130/vns-0025-0010 (Accessed August 18, 2026)

[66] Yang M Bian F Feng X Li L Huang H Liu L. GPX4 Promotes optic nerve regeneration and retinal ganglion cell neuroprotection. Mol Ther. (2026) 34:1938–44. 10.1016/j.ymthe.2025.12.057 41485051 PMC12831598

[67] Tang L Xu GT Zhang JF. Inflammation in diabetic retinopathy: possible roles in pathogenesis and potential implications for therapy. Neural Regen Res. (2023) 18:976–82. 10.4103/1673-5374.355743 36254977 PMC9827774

[68] He W Tang P Lv H. Targeting oxidative stress in diabetic retinopathy: mechanisms, pathology, and novel treatment approaches. Front Immunol. (2025) 16:1571576. 10.3389/fimmu.2025.1571576 40589740 PMC12207546

[69] Liu S Chen C Ning J Ding P Chen Y Xie T. Association between early inflammation and neurodegeneration in diabetic retinopathy: a narrative review. VNS. (2025) 42:e012. 10.48130/vns-0025-0012

[70] Ma H Wang X Zhang W Li H Zhao W Sun J. Melatonin suppresses ferroptosis induced by high glucose via activation of the Nrf2/HO-1 signaling pathway in type 2 diabetic osteoporosis. Oxid Med Cell Longevity. (2020) 2020:9067610–18. 10.1155/2020/9067610 PMC7732386 33343809

[71] Trudeau K Molina AJA Roy S. High glucose induces mitochondrial morphology and metabolic changes in retinal pericytes. Invest Ophthalmol Vis Sci. (2011) 52:8657–64. 10.1167/iovs.11-7934 21979999 PMC3230288

[72] Njie-Mbye YF Kulkarni-Chitnis M Opere CA Barrett A Ohia SE. Lipid peroxidation: pathophysiological and pharmacological implications in the eye. Front Physiol. (2013) 4:366. 10.3389/fphys.2013.00366 24379787 PMC3863722

[73] Al-Shabrawey M Rojas M Sanders T Behzadian A El-Remessy A Bartoli M. Role of NADPH oxidase in retinal vascular inflammation. Invest Ophthalmol Vis Sci. (2008) 49:3239–44. 10.1167/iovs.08-1755 18378574 PMC3798055

[74] Rojas M Zhang W Xu Z Lemtalsi T Chandler P Toque HA. Requirement of NOX2 expression in both retina and bone marrow for diabetes-induced retinal vascular injury. PLoS One. (2013) 8:e84357. 10.1371/journal.pone.0084357 24358357 PMC3866146

[75] Hao XD Xu WH Zhang X Xue J. Targeting ferroptosis: a novel therapeutic strategy for the treatment of retinal diseases. Front Pharmacol. (2024) 15:1489877. 10.3389/fphar.2024.1489877 39539617 PMC11557320

[76] Li L Dai Y Ke D Liu J Chen P Wei D. Ferroptosis: new insight into the mechanisms of diabetic nephropathy and retinopathy. Front Endocrinol (Lausanne). (2023) 14:1215292. 10.3389/fendo.2023.1215292 37600716 PMC10435881

[77] Gao Y Zhao W Chai Q Ye C. ELAVL1 Stabilizes HMOX1 mRNA to drive ferroptosis in diabetic retinopathy. Diabetes Metab Syndr Obes. (2025) 18:3687–703. 10.2147/DMSO.S536325 41084510 PMC12515415

[78] Liu D Zheng Z Chen Z Guo R Weng J Huang S. Ferroptosis in müller cells under hyperglycemia: mechanisms and therapeutic implications for diabetic retinopathy-associated optic neuroinflammation. Int Ophthalmol. (2025) 45:302. 10.1007/s10792-025-03681-5 40690064

[79] Yang Y Zou H. Research progress on Nrf2 intervention in the treatment of diabetic retinopathy. Front Endocrinol (Lausanne). (2025) 16:1587231. 10.3389/fendo.2025.1587231 40469432 PMC12133500

[80] Chaitanuwong P Moss HE. Optic neuritis: a comprehensive review of current therapies and emerging treatment strategies. Front Neurol. (2025) 16:1605075. 10.3389/fneur.2025.1605075 40606143 PMC12214357

[81] Jian J Wei J. Ferroptosis: a new pathway in the interaction between gut microbiota and multiple sclerosis. Front Biosci (Landmark Ed). (2025) 30:26265. 10.31083/FBL26265 39862079

[82] Rubinsztein DC. Immunoproteasome-dependent neuronal ferroptosis in multiple sclerosis. Cell Res. (2026) 36:1–2. 10.1038/s41422-025-01162-z 40721537 PMC12765852

[83] Hoshino T Yamakado H Takahashi R Matsuzawa SI. Susceptibility to erastin-induced ferroptosis decreases during maturation in a human oligodendrocyte cell line. FEBS Open Bio. (2020) 10:1758–64. 10.1002/2211-5463.12923 32608563 PMC7459400

[84] Kłodnicka K Januszewski J Forma A Pająk W Teresińska B Baj J. Iron in multiple sclerosis - from pathophysiology to disease progression - a narrative literature review. Acta Neurobiol Exp (Wars). (2025) 85:75–93. 10.55782/ane-2025-2680 40742010

[85] Lin Z Zou S Wen K. The crosstalk of CD8+ T cells and ferroptosis in cancer. Front Immunol. (2023) 14:1255443. 10.3389/fimmu.2023.1255443 38288118 PMC10822999

[86] del Amo EM Rimpelä AK Heikkinen E Kari OK Ramsay E Lajunen T. Pharmacokinetic aspects of retinal drug delivery. Prog Retin Eye Res. (2017) 57:134–85. 10.1016/j.preteyeres.2016.12.001 28028001

[87] De Clerck K De Smedt S Remaut K Peynshaert K. Toward successful retinal drug delivery after intravitreal injection: current strategies to overcome the inner limiting membrane. J Control Release. (2025) 384:113849. 10.1016/j.jconrel.2025.113849 40393532

[88] Wang Y Rajala A Rajala RVS. Lipid nanoparticles for ocular gene delivery. J Funct Biomater. (2015) 6:379–94. 10.3390/jfb6020379 26062170 PMC4493518

[89] Peynshaert K Devoldere J Minnaert AK De Smedt SC Remaut K. Morphology and composition of the inner limiting membrane: species-specific variations and relevance toward drug delivery research. Curr Eye Res. (2019) 44:465–75. 10.1080/02713683.2019.1565890 30638413

[90] Song D Zhao L Li Y Hadziahmetovic M Song Y Connelly J. The oral iron chelator deferiprone protects against systemic iron overload-induced retinal degeneration in hepcidin knockout mice. Invest Ophthalmol Vis Sci. (2014) 55:4525–32. 10.1167/iovs.14-14568 24970260 PMC4106252

[91] Entezari S Haghi SM Norouzkhani N Sahebnazar B Vosoughian F Akbarzadeh D. Iron chelators in treatment of iron overload. J Toxicol. (2022) 2022:4911205–18. 10.1155/2022/4911205 35571382 PMC9098311

[92] Cabezón I Manich G Martín-Venegas R Camins A Pelegrí C Vilaplana J. Trafficking of gold nanoparticles coated with the 8D3 anti-transferrin receptor antibody at the mouse blood-brain barrier. Mol Pharm. (2015) 12:4137–45. 10.1021/acs.molpharmaceut.5b00597 26440359

[93] Dong S Pan Z Chang M Zhou J Zhang M Chen Y. Lipid nanoparticles-mediated mRNA delivery to the eye affected by ionizable cationic lipid. Mol Pharm. (2025) 22:3297–307. 10.1021/acs.molpharmaceut.5c00248 40331524

[94] Laughter MR Ammar DA Bardill JR Pena B Kahook MY Lee DJ. A self-assembling injectable biomimetic microenvironment encourages retinal ganglion cell axon extension *in vitro*. ACS Appl Mater Interfaces. (2016) 8:20540–8. 10.1021/acsami.6b04679 27434231 PMC5752433

[95] Li Z Wang W Zhu L Wang Z Liu C Sun Y. Reprogramming retinal microglia polarization by efferocytosis-mimicking nanoparticles for ameliorating diabetic retinopathy. ACS Appl Mater Interfaces. (2025) 17:29210–23. 10.1021/acsami.5c03170 40329689

[96] Lu Y Zhu F Zhou X Li Y Rong G Liu N. A supramolecular deferoxamine-crisaborole nanoparticle targets ferroptosis, inflammation, and oxidative stress in the treatment of retinal ischemia/reperfusion injury. Nano Lett. (2025) 25:1058–66. 10.1021/acs.nanolett.4c05012 39670541

[97] Liang Y Tian Y Liu J Lei P Sun X Zhang H. Smart bioorthogonal catalytic factory for glaucoma therapy. Nano Lett. (2025) 25:5502–11. 10.1021/acs.nanolett.5c00607 40102044

[98] Tang Y Han Y Zhao J Lv Y Fan C Zheng L. A rational design of metal-organic framework nanozyme with high-performance copper active centers for alleviating chemical corneal burns. Nanomicro Lett. (2023) 15:112. 10.1007/s40820-023-01059-9 37121915 PMC10149557

[99] Wang LH Huang CH Lin IC. Advances in neuroprotection in glaucoma: pharmacological strategies and emerging technologies. Pharmaceuticals (Basel). (2024) 17:1261. 10.3390/ph17101261 39458902 PMC11510571

[100] Bigot K Gondouin P Bénard R Montagne P Youale J Piazza M. Transferrin non-viral gene therapy for treatment of retinal degeneration. Pharmaceutics. (2020) 12:836. 10.3390/pharmaceutics12090836 32882879 PMC7557784

[101] Youale J Bigot K Jaworski T Lebon C Françon A Delaunay K. Transferrin is a drug candidate for the treatment of dry age-related macular degeneration (AMD). Cell Death Dis. (2025) 16:692. 10.1038/s41419-025-07950-0 41053100 PMC12501284

[102] Pan Y Wang X Liu X Shen L Chen Q Shu Q. Targeting ferroptosis as a promising therapeutic strategy for ischemia-reperfusion injury. Antioxidants (Basel). (2022) 11:2196. 10.3390/antiox11112196 36358568 PMC9686892

[103] Liang FG Zandkarimi F Lee J Axelrod JL Pekson R Yoon Y. OPA1 Promotes ferroptosis by augmenting mitochondrial ROS and suppressing an integrated stress response. Mol Cell. (2024) 84:3098–3114.e6. 10.1016/j.molcel.2024.07.020 39142278 PMC11373561

[104] Hoelzgen F Nguyen TTP Klukin E Boumaiza M Srivastava AK Kim EY. Structural basis for the intracellular regulation of ferritin degradation. Nat Commun. (2024) 15:3802. 10.1038/s41467-024-48151-1 38714719 PMC11076521

[105] Sun W-J An X-D Zhang Y-H Tang S-S Sun Y-T Kang X-M. Autophagy-dependent ferroptosis may play a critical role in early stages of diabetic retinopathy. World J Diabetes. (2024) 15:2189–202. 10.4239/wjd.v15.i11.2189 39582563 PMC11580571

[106] Wu K Zhao W Hou Z Zhang W Qin L Qiu J. Ferritinophagy: multifaceted roles and potential therapeutic strategies in liver diseases. Front Cell Dev Biol. (2025) 13:1551003. 10.3389/fcell.2025.1551003 40070880 PMC11893559

[107] Fan BY Pang YL Li WX Zhao CX Zhang Y Wang X. Liproxstatin-1 is an effective inhibitor of oligodendrocyte ferroptosis induced by inhibition of glutathione peroxidase 4. Neural Regen Res. (2021) 16:561–6. 10.4103/1673-5374.293157 32985488 PMC7996026

[108] Scarpellini C Klejborowska G Lanthier C Hassannia B Vanden Berghe T Augustyns K. Beyond ferrostatin-1: a comprehensive review of ferroptosis inhibitors. Trends Pharmacol Sci. (2023) 44:902–16. 10.1016/j.tips.2023.08.012 37770317

[109] Carlson BA Tobe R Yefremova E Tsuji PA Hoffmann VJ Schweizer U. Glutathione peroxidase 4 and vitamin E cooperatively prevent hepatocellular degeneration. Redox Biol. (2016) 9:22–31. 10.1016/j.redox.2016.05.003 27262435 PMC4900515

[110] Esposito G Cosimi P Balzamino BO Bruno M Squitti R Dinice L. Ceruloplasmin and ferritin changes in ocular fluids from patients with vitreoretinal diseases: relation with neuroinflammation and drusen formation. Int J Mol Sci. (2025) 26:6307. 10.3390/ijms26136307 40650085 PMC12250406

[111] Babighian S Gattazzo I Zanella MS Galan A D'Esposito F Musa M. Nicotinamide: bright potential in glaucoma management. Biomedicines. (2024) 12:1655. 10.3390/biomedicines12081655 39200120 PMC11352092

[112] Shukla AG Cioffi GA John SWM Wang Q Liebmann JM. American Glaucoma society and American academy of ophthalmology. American glaucoma society-American academy of ophthalmology position statement on nicotinamide use for glaucoma neuroprotection. Ophthalmol Glaucoma. (2025) 8:112–6. 10.1016/j.ogla.2025.01.002 39800263

[113] Li J Jia YC Ding YX Bai J Cao F Li F. The crosstalk between ferroptosis and mitochondrial dynamic regulatory networks. Int J Biol Sci. (2023) 19:2756–71. 10.7150/ijbs.83348 37324946 PMC10266069

[114] Kowluru RA Kowluru A Veluthakal R Mohammad G Syed I Santos JM. TIAM1-RAC1 Signalling axis-mediated activation of NADPH oxidase-2 initiates mitochondrial damage in the development of diabetic retinopathy. Diabetologia. (2014) 57:1047–56. 10.1007/s00125-014-3194-z 24554007 PMC10317023

[115] Zhan P Lu X Li Z Wang WJ Peng K Liang NN. Mitoquinone alleviates bleomycin-induced acute lung injury via inhibiting mitochondrial ROS-dependent pulmonary epithelial ferroptosis. Int Immunopharmacol. (2022) 113:109359. 10.1016/j.intimp.2022.109359 36288647

[116] Automated cup-to-disc ratio quantification via color fundus photography for chronic glaucoma screening - PubMed. Available online at: https://pubmed.ncbi.nlm.nih.gov/41152744/ (Accessed August 18, 2026) 10.1186/s12880-025-01981-x PMC12560540 41152744

[117] Yang WH Xu YW Sun XH. Expert workgroup of guidelines for glaucoma imaging classification, annotation, and quality control for artificial intelligence applications. Guidelines for glaucoma imaging classification, annotation, and quality control for artificial intelligence applications. Int J Ophthalmol. (2025) 18:1181–96. 10.18240/ijo.2025.07.01 40688788 PMC12207309

[118] Sato K Saigusa D Kokubun T Fujioka A Feng Q Saito R. Reduced glutathione level in the aqueous humor of patients with primary open-angle glaucoma and normal-tension glaucoma. NPJ Aging. (2023) 9:28. 10.1038/s41514-023-00124-2 37990002 PMC10663551

[119] Chang D Sha Q Zhang X Liu P Rong S Han T. The evaluation of the oxidative stress parameters in patients with primary angle-closure glaucoma. PLoS One. (2011) 6:e27218. 10.1371/journal.pone.0027218 22096540 PMC3214032

[120] Seven E Tekin S. Comparative analysis of oxidative stress biomarkers in 50 primary open-angle glaucoma patients. Med Sci Monit. (2025) 31:e948665. 10.12659/MSM.948665 40542493 PMC12188943

[121] Expert consensus on the application of artificial intelligence in neuro-ophthalmic diseases. Available online at: https://www.maxapress.com/article/doi/10.48130/vns-0025-0018 (Accessed August 18, 2026).

[122] Huang W Ogbuji R Zhou L Guo L Wang Y Kopell BH. Motoric impairment versus iron deposition gradient in the subthalamic nucleus in Parkinson's Disease. J Neurosurg. (2021) 135:284–90. 10.3171/2020.5.JNS201163 32764171

[123] Gu J Ruan J Li Z Fu H Shi Q Zhu J. Cadmium induces ferroptosis in B cells via ATP6V0A1-upregulated lysosomal ferritinophagy: insights from murine transcriptomics and human cellular models. Free Radic Biol Med. (2026) 242:138–49. 10.1016/j.freeradbiomed.2025.10.287 41161393

[124] Yin J Zhan J Hu Q Huang S Lin W. Fluorescent probes for ferroptosis bioimaging: advances, challenges, and prospects. Chem Soc Rev. (2023) 52:2011–30. 10.1039/d2cs00454b 36880388

[125] Yang W Zheng Y Wang S. Editorial: imaging in the diagnosis and treatment of eye diseases. Front Ophthalmol (Lausanne). (2026) 6:1899308. 10.3389/fopht.2026.1899308 42395831 PMC13322848

[126] Kim BK Goncharov T Archaimbault SA Roudnicky F Webster JD Westenskow PD. RIP1 Inhibition protects retinal ganglion cells in glaucoma models of ocular injury. Cell Death Differ. (2025) 32:353–68. 10.1038/s41418-024-01390-7 39448868 PMC11802773

[127] Yang Y Sun X. Retinal ganglion cell death in glaucoma: advances and caveats. Curr Eye Res. (2023) 48:1–10. 10.1080/02713683.2022.2068182 35614865

